# Trends in type 2 diabetes incidence and BMI at diagnosis by age, sex, and ethnicity, 2011–2024: a population-based analysis of the English National Diabetes Audit

**DOI:** 10.1016/j.lanepe.2026.101764

**Published:** 2026-07-22

**Authors:** Naomi Holman, Jackie O’Keefe, Edward Gregg, Garry Daniel Tan, Kamlesh Khunti, Jonathan Valabhji, Chirag Bakhai, Clare Hambling, Bob Young, Shivani Misra

**Affiliations:** a School of Public Health, Imperial College, London, UK; b School of Population Health, RCSI University of Medicine and Health Sciences, Dublin, Ireland; c Oxford Centre for Diabetes, Endocrinology & Metabolism, Oxford University Hospitals, Oxford, UK; d NIHR Oxford Biomedical Research Centre, UK; e Diabetes UK, London, UK; f Diabetes Research Centre, Leicester General Hospital, University of Leicester, Leicester, UK; g Division of Metabolism, Digestion and Reproduction, Faculty of Medicine, Imperial College London, London, UK; h Chelsea & Westminster Hospital NHS Foundation Trust, London, UK; i Diabetes Programme, NHS England, London, UK; j Bedfordshire Luton and Milton Keynes Integrated Care Board, Luton, UK; k Norfolk & Waveney Integrated Care Board, Norwich, UK; l Litcham Health Centre, King’s Lynn, UK; m Department of Diabetes & Endocrinology, Imperial College Healthcare NHS Trust, London, UK

**Keywords:** Early-onset type 2 diabetes, Incidence, Epidemiology, Ethnicity, Type 2 diabetes, Young type 2 diabetes

## Abstract

**Background:**

Early-onset type 2 diabetes is associated with aggressive disease progression, long-term health burden and reduced life expectancy. Trends in incidence have not been comprehensively described.

**Methods:**

Using data from the English National Diabetes Audit, age-standardised incidence rates for adults with type 2 diabetes were calculated by age, sex, and ethnicity between 2011 and 2024. Poisson regression models were created to study incidence over time. Annual change in median BMI within 26 weeks of diagnosis were calculated.

**Findings:**

Age-standardised incidence increased among adults aged 20–29 years (RR 1.021 [95% CI 1.017–1.026] per year in males, 1.030 [1.026–1.034] in females) and 30–39 years (1.013 [1.012–1.015] in males, 1.03 [1.028–1.032] in females). Incidence increased minimally in males aged 40–59 years (1.006 [1.005–1.006]), was rising in females (1.018 [1.017–1.019]) and declined in males and females aged 60–79 years (0.983 [0.983–0.984] in males, 0.985 in females 0.985 [0.984–0.985]). Increasing incidence in young adults were observed in all sex and ethnicity subgroups except Asian males (20–39-years) but highest rates were in White females (30–39-years) and Black females (20–29). BMI at diagnosis increased in all subgroups over time but was highest in younger adults and higher in females than males. The annual change in younger adults was double that of older (20–29 year-old-males +0.17 kg/m^2^/year [0.08–0.26] and females +0.17 [0.09–0.26]; 30–39-year-old males +0.21 [0.18–0.24] and females +0.20 [0.16–0.24]; 40–59 year-old-males +0.06 [0.04–0.07] and females +0.09 [0.06–0.12]; 60–79 year old males +0.08 [0.06–0.09] and females +0.11 [0.08–0.13]).

**Interpretation:**

Since 2011, incidence of type 2 diabetes has risen significantly in young adults across most ethnicity and sex subgroups (especially females) despite minimal changes or declining rates at older ages. This trend parallels greater increases in BMI-at-diagnosis in young people, highlighting a shift in disease burden and the urgent need for earlier prevention, and focused obesity interventions in early-life.

**Funding:**

Wellcome Trust.

**Research in context**

**Evidence before this study**

We conducted a search of PubMed from database inception to 16th November 2025. We searched for existing reviews and original research studies related to the epidemiology of type 2 diabetes in younger populations or with age-stratified analyses of incidence. We used the search terms “type 2 diabetes”, “early-onset”, “youth-onset”, “epidemiology “, “incidence” and “age-standardised”, “ethnicity”, “sex”. We prioritised population-based epidemiological studies and original data articles examining incidence and/or provided estimates by age, sex and ethnicity.

Early-onset type 2 diabetes follows a more aggressive course, is associated with significant loss of life expectancy and is likely to place a substantial long-term burden on health services despite lower absolute case numbers than in older populations. Incidence estimates for early-onset type 2 diabetes in adults aged 20–39 years are poorly studied and have shown a mixed pattern, with some analyses reporting rising rates while others demonstrate stability or no increase across regions. Overall type 2 diabetes trend estimates are predominantly driven by later-onset disease, which accounts for the majority of incident cases and may obscure important age-specific divergences. A comprehensive analysis examining trends across the full adult age spectrum, sex and ethnicity has not been undertaken to frame changes in early-onset incidence against concurrent shifts in older age groups, nor have trends in BMI at diagnosis been explored.

**Added value of this study**

Stratifying incidence by age, our analysis of English National Diabetes data with population coverage, demonstrates divergent temporal patterns, with rising incidence in young adults aged 20–39 years occurring alongside declining or plateauing rates in older age groups.

For the first time we identify marked ethnic variation across all age strata. Age-standardised incidence remains higher among Asian and Black populations in every age group analysed. Notably, we observe higher incidence in females aged 20–29 years across all ethnicities, whereas males predominate in all other age strata. The increasing trend in young adult incidence appears to be driven predominantly by females from all ethnic groups studied and by White and Black males. All ethnic groups aged 60–79 year’s show a declining trend over the study period.

BMI at diagnosis (not previously explored at scale) demonstrate substantial increases at diagnosis over the study period, with the annual change per year almost double in younger age groups compared to older, equating to a an ∼2.5 kg/m^2^ in younger adults over the study period. These findings suggest that younger individuals may be particularly susceptible to environmental drivers of obesity and early-onset type 2 diabetes.

**Implications of all the available evidence**

The rising incidence of type 2 diabetes among younger adults has important clinical and public health implications. Although incidence remains highest among Asian and Black groups, the increase in younger adults is also evident among White individuals, suggesting that early-onset disease is becoming more widespread rather than confined to traditionally high-risk ethnic groups. The higher BMI observed at diagnosis in younger cohorts further compounds risk and supports the need for treatment strategies that prioritise weight reduction and consider remission early in the disease pathway. From a prevention perspective, these findings suggest that current approaches to obesity prevention are not reaching younger adults effectively. Earlier and more intensive interventions addressing diet, physical activity, and the wider determinants of severe obesity are needed.

## Introduction

Early-onset type 2 diabetes, defined as a diagnosis below the age of 40-years is rising in prevalence globally.^1^^,^^2^ An early diagnosis is concerning as, relative to typical later-onset presentations, there is an association with earlier mortality,^3^ faster progression to microvascular and macrovascular complications^4^^,^^5^ and in women, compared to those with type 1 diabetes, worse pregnancy outcomes.^6^

The epidemiology of early-onset type 2 diabetes has been comprehensively characterised in youth (<20 years), among whom incidence rates increased significantly across all ethnicities between 2002 and 2018 in the United States, with markedly higher rates observed in minority ethnic populations.^1^ However, there are few studies of incidence in young adults with early-onset type 2 diabetes (aged 18–39 years),^7^ who share an elevated risk of adverse outcomes with youth but represent a substantially larger proportion of the early-onset population. A Swedish study reported rising aged-standardised incidence in 23–39-year-olds from 2006–2021^8^ and in another multi-country study, estimated incidence of type 2 diabetes significantly increased in 15–39-year-olds across 204 countries between 1990 to 2019.^2^ Another study of type 2 diabetes incidence in people aged 15–39 across eight regions reported a mixed picture, with East Asian regions showing increasing incidence, but in the six countries with predominantly white European populations, two observed an increase in incidence, two no change and two a decrease.^9^ Therefore, the incidence of type 2 diabetes in young adults shows a mixed pattern and trends in the incidence of early-onset type 2 diabetes have not been studied by ethnic group outside of the US.

In England, the cross-sectional prevalence of early-onset type 2 diabetes has shown to disproportionately affect young adults from minority ethnicities.^7^ However, trends in incidence across ethnicities and age strata have not been examined but are needed to understand the changing epidemiology of type 2 diabetes.

We analysed trends in the incidence of type 2 diabetes in England using the National Diabetes Audit (NDA) by age, sex and ethnicity between 2011 and 2024 to place emerging patterns in young adults within the broader context. Recognising that obesity is the major driver of early-onset type 2 diabetes, we also examined changes in body mass index (BMI) at diagnosis by age, sex and ethnicity.

## Methods

We undertook a population analysis of trends in age-standardised incidence of type 2 diabetes using several national datasets.

### Data sources

The NDA has collated data annually on people with a valid code for diagnosed diabetes, registered with a primary care or specialist healthcare provider in England since 2003.^10^ Since 2017 there has been virtually complete participation in the audit (>99% population coverage).^10^

Hospital Episode Statistics (HES) is a record of all NHS activity in hospitals and includes NHS number and demographic details^11^ and was used to supplement data on ethnicity if not included in the NDA.

The Office for National Statistics produces annual population estimates of the population of England by age and sex based on birth rates, death rates and migration but not ethnicity.^12^^,^^13^ Ethnicity-specific population denominators stratified by age and sex were only available from decennial Census data. We used data from the 2011 and 2021 Censuses to identify the proportion of the population in each ethnic group by sex across five-year age bands.^12^^,^^13^ We used linear interpolation between census years to derive interim estimates and assumed that the proportions changed at a constant rate and continued at the same rate to 2024. These proportions were combined with the Office for National Statistics population estimates to create population estimates by five-year age bands, sex and ethnicity for each of the years from 2011 to 2024.

### Definitions

#### Study period

We extracted all data on people coded with type 2 diabetes with a diagnosis date from 2009 to 2022 registered with a primary care provider in England from the NDA.

#### Identification of type 2 diabetes

Type 2 diabetes was identified from the diagnostic codes collated by the NDA from primary care records and specialist diabetes services. Where a specialist diabetes service (from HES) had reported one or more diagnostic codes, the latest reported type of diabetes was used. Where no data had been provided by a specialist diabetes service, the latest type recorded in the general practice electronic health record was used. Sex was male or female and used as recorded in the NDA.

#### Ethnicity

Where a valid ethnicity had been recorded in the NDA dataset the latest valid ethnicity was allocated to the individual. Where a valid ethnicity was not recorded in the NDA dataset, the latest valid ethnicity code recorded in outpatient or inpatient HES episodes between 1st April 2000 and 31st March 2024 was allocated to that individual. Data for White, Asian and Black individuals were extracted, representing the three largest ethnicities in England.

#### Incidence

Since the NDA collection started there has been variation in the time lag between the end of the audit period and data extraction. In recent years, extraction of data occurred within three months, but historically the time-lag was up to 15 months. The impact of this lag is that in earlier audit years, people who were no longer registered with their healthcare provider at the time of data extraction would not appear in the dataset. To standardise our analysis across the study period we defined incidence as type 2 diabetes that had been diagnosed for at least 18 months between the end of the audit period and the date of data extraction (Supplementary Fig. S1). For example, the incidence of diagnosed type 2 diabetes for 2024 includes individuals diagnosed with type 2 diabetes between 1st January 2022 and 31st December 2022, who were alive on 1st July 2024.

#### BMI

Up until 2016/17 the NDA collected the latest BMI measurement within the audit period and from 2017/18 onwards all BMI measurements recorded were extracted. To optimise ascertainment while retaining relevance to BMI at presentation, the earliest BMI measured within 26 weeks of diagnosis was identified from the NDA data.

### Statistical methods

The coverage of the NDA in the data collections included in this analysis ranged from 54.9% to 99.2% of general practices in England (>90% since 2016). To account for this variation the number of incident cases for each year were divided by the proportion of general practices contributing to estimate the number of people meeting these criteria across all practices in England. This adjusted count is used as the numerator for all incidence rates calculated.

Directly age standardised incidence rates using the total general population in 2023 in England as the standard population were calculated by sex for the ages 20–29 years, 30–39 years, 40–59 years, and 60–79 years for each year from 2011 to 2024. Z-tests were used to assess differences in age standardised rates between females and males, ANOVA and Kruskal–Wallis tests were used to test differences across age groups or over each of the 14 years of the study.

Poisson regression models were created to assess change in incidence over time. Separate models were run for ages 20-to-29 years, 30-to-39 years, 40-to-59 years and 60-to-79 years. To assess change in incidence across the whole population, the explanatory variables were age in five-year bands, sex and year with sex by year as an interaction term to assess whether change in incidence over time varied between males and females. The inclusion of year as a continuous variable assumes that the rate of change in incidence is uniform across the time period studied.

To assess whether incidence changed by ethnic group models with five-year age bands, sex, ethnic group, year and year by ethnic group by sex as an interaction term as explanatory variables were created for the four age groups.

The proportion of people with a BMI recorded within 26-weeks after diagnosis varied from 57.9% in females aged 20-to-29 years old in 2012 to 74.7% in males aged 40-to 59-years old in 2021 (Supplementary Table S7). Missing data were imputed using predictive mean matching (using age, sex, ethnicity and quintile of social deprivation) from the cohort with the same year of diagnosis. 95% confidence intervals were calculated using the Bryar’s method. We calculated the annual change in median BMI stratified by age, sex and ethnicity in both absolute and relative terms and tested for significance using Kruskal–Wallis tests.

#### Sensitivity analyses

To assess the impact of the definition of requiring incidence to be at least 18-months duration, we conducted a sensitivity analysis during later years when there was a minimal lag between the end of the audit period and data extraction. Poisson regression models were created to compare the rate ratio per additional year for the two definitions for the time period 2018 to 2024.

To address potential misclassification of type 2 diabetes a sensitivity analysis excluding individuals initiated on insulin <6-months of diagnosis was performed. Rate ratios calculated using Poisson models with the same structure as the primary analysis were used to assess whether the refined definition of type 2 diabetes altered trends observed. This was restricted to latter years of the study with available prescription data in the NDA (2018–2024).

To assess the scale of the reduction of diagnoses of type 2 diabetes that occurred during the first lockdown in the COVID19 pandemic the incidence rates for 2022 to 2024 were predicted using Poisson regression models with the same structure described using data for 2011 to 2020 and compared to actual cumulative incidence rates for the same time period.

### Information governance

NDA data is collected and used in line with NHS England’s purposes as required under the statutory duties outlined in the NHS Act 2006 and Health and Social Care Act 2012. As the data are collected for audit purposes, individual level patient consent is not required for patients accessing services in England. There is controlled access to data held on secure data environments entirely within the NHS England infrastructure. Data is processed for specific purposes only, including operational functions, service evaluations and service improvement. The present analysis was undertaken to support a service improvement initiative in England for those with type 2 diabetes aged under 40 years old. Ethics committee approval is not required for these specific purposes. All numbers taken from the NDA are rounded to the nearest 5 to protect individuals’ confidentiality.

#### Role of the funding source

The funder had no role in study design, data collection, data analysis or interpretation.

## Results

The number of incident cases of type 2 diabetes aged 20-to-79 years recorded in the NDA increased from 56,980 to 96,455 for females and 80,000 to 122,720 for males between 2011 and 2024 (Supplementary Table S1). Adjustment to compensate for the coverage of the NDA changed these figures to 72,300 [95% CI 71,680–73,310] and 101,520 [100,640–102,930] in 2011 and 98,190 [97,885–98,495] and 124,930 [124,540–125,315] in 2024 respectively (Supplementary Table S2).

### Age standardised incidence by age, sex and ethnicity

**Fig. 1:**
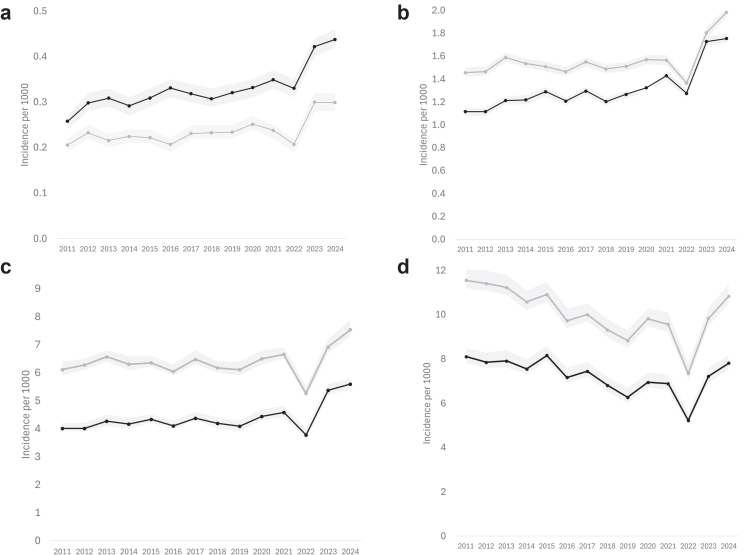
Age-standardised incidence of type 2 diabetes by age and sex. Panel a) individuals aged 20–29 years old, b) 30–39 years old, c) 40–59 years old and d) 60–79 years old. Grey lines denote males and black lines are females. Shaded area denotes 95% confidence intervals. Raw data can be viewed in supplementary table S4.

In people aged 20-to-29 years, age standardised incidence of type 2 diabetes was higher in females than in males (0.44 [95% CI 0.42–0.46] vs. 0.30 [0.28–0.32] per 1000 person-years in 2024) but the sex differences were reversed in all other age groups; 30–39 years (1.76 [1.72–1.80] in females vs. 1.98 [1.94–2.03] in males per 1000 person-years); 40–59 years (7.5 [7.3–7.9] vs. 5.6 [5.4–5.8] per 1000 person-years) and 60–79 years (10.8 [10.5–11.4] vs. 7.8 [7.5–8.2] per 1000 person-years) age groups (Fig. 1, Supplementary Table S3 (crude incidence) and 4 (age-standardised)).

**Fig. 2:**
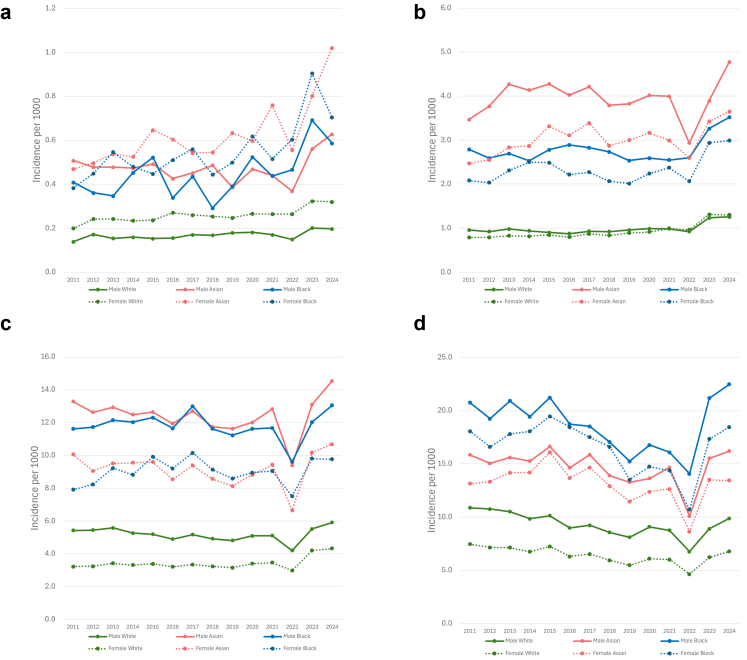
Age-standardised incidence of type 2 diabetes by age, ethnicity and sex. Panel a) individuals aged 20–29 years old, b) 30–39 years old, c) 40–59 years old and d) 60–79 years old. Dotted lines are females and solid lines are males. Confidence intervals for the incidence rates shown can be viewed in Supplementary Table S5.

Across all age groups and years of study, the age-standardised incidence of type 2 diabetes was higher in South Asian and Black males and females compared to White males and females (Fig. 2, Supplementary Table S5).

Median age-at-diagnosis for males and females was broadly similar across the study period within each age category (Supplementary Table S6).

### Trends in incidence by age and sex

Between 2011 and 2024, annual change in incidence varied by age group and sex. Among younger adults, incidence increased in both males and females, with steeper increases observed in females (Table 1). In individuals aged 20–29 years, incidence increased by 2.1% per year in males (Rate Ratio (RR) per additional year 1.021, 95% CI 1.017–1.026) and 3.0% per year in females (RR 1.030 [1.026–1.034]); interaction term sex x year p = 0.004.

**Table 1:** Incidence rate ratios by age group, sex and ethnicity analysing trends in incidence of type 2 diabetes between 2011 and 2024, with 95% confidence intervals.

Sex	Ethnic group	Rate ratio/p-value	20–29	30–39	40–59	60–79
Males	All	RR	1.021 (1.017–1.026)	1.013 (1.012–1.015)	1.006 (1.005–1.006)	0.983 (0.983–0.984)
Females		RR	1.030 (1.026–1.034)	1.030 (1.028–1.032)	1.018 (1.017–1.019)	0.985 (0.984–0.985)
		p value	0.004	<0.0001	<0.0001	<0.0001
Combined	White	RR	1.022 (1.019–1.026)	1.028 (1.026–1.03)	1.004 (1.003–1.004)	0.98 (0.98–0.981)
	Asian	RR	1.028 (1.021–1.034)	1.008 (1.005–1.01)	1 (0.999–1.002)	0.988 (0.986–0.989)
	Black	RR	1.041 (1.03–1.052)	1.014 (1.009–1.019)	1.004 (1.002–1.006)	0.986 (0.983–0.988)
	White	p value	–	–	–	–
	Asian	p value	0.118	<0.0001	<0.0001	<0.0001
	Black	p value	0.002	<0.0001	0.439	<0.0001
Males	White	RR	1.018 (1.012–1.024)	1.018 (1.015–1.020)	0.997 (0.996–0.998)	0.981 (0.980–0.981)
	Asian	RR	1.006 (0.997–1.015)	1.001 (0.998–1.005)	1.005 (0.988–1.022)	0.989 (0.987–0.992)
	Black	RR	1.035 (1.018–1.052)	1.012 (1.005–1.018)	1.001 (0.999–1.004)	0.990 (0.986–0.993)
Females	White	RR	1.025 (1.021–1.030)	1.038 (1.035–1.041)	1.014 (1.013–1.015)	0.980 (0.979–0.981)
	Asian	RR	1.044 (1.036–1.053)	1.016 (1.012–1.020)	1.000 (0.999–1.002)	0.986 (0.984–0.988)
	Black	RR	1.045 (1.031–1.059)	1.016 (1.010–1.023)	1.008 (1.005–1.011)	0.983 (0.979–0.986)
Males	White	p value	0.045	<0.001	<0.001	0.162
	Asian	p value	<0.001	0.041	<0.001	0.002
	Black	p value	0.926	0.002	<0.001	0.001
Females	White	p value	–	–	–	–
	Asian	p value	<0.001	<0.001	<0.001	<0.001
	Black	p value	0.012	<0.001	<0.001	0.452

p-values for interaction terms testing trends whether trends differ between groups specified.

A similar pattern was observed in those aged 30–39 years although females showed a 2-fold higher increase per annum; males RR 1.013 [1.012–1.015]; females RR 1.030 [1.028–1.032]; sex x year p < 0.0001.

The pattern of higher annual increases in incidence in females was also observed in 40–59-year-olds; females showing a 1.8% increase per annum (RR 1.018 [1.017–1.019]; sex x year p < 0.0001). Males also showed an increasing trend in this age group, however the annual increase was significantly lower; males RR 1.006 [1.005–1.006].

In contrast to younger age groups, incidence showed a declining trend in adults aged 60–79 years with no relevant difference in the rate of change per year observed in males or females (males RR 0.983 [0.983–0.984]; females RR 0.985 [0.984–0.985]; sex x year p < 0.0001).

### Sensitivity analyses

A sensitivity analysis, restricted to the last six years of the study period (time-lag between audit end and data collection was <6 months), showed no difference in incidence rate ratio by age and sex subgroup (Supplementary Table S7), supporting that the longer lag in the primary analysis did not alter trends in incidence over time.

A very low proportion of individuals coded with a type 2 diabetes diagnosis were exclusively treated with insulin (<1.5% across all subgroups (Supplementary Table S8)). Higher proportions received an insulin prescription within six months of diagnosis (Supplementary Table S9), particularly younger adults (e.g., 10.1% in males aged 20–29 years and 6.5% in those aged 30–39 years, compared with 4.0% aged 40–59 years and 2.7% in 60–79 years). Excluding individuals prescribed insulin within six months of diagnosis did not alter the annual change in incidence in any age and sex group (Supplementary Table S10).

There was a clear reduction in the incidence of type 2 diabetes across all subgroups in 2022 reflecting a fall in diagnoses in 2020 associated with the first lockdown of the COVID-19 pandemic. The actual cumulative incidence rate for 2022 to 2024 exceeds the predicted cumulative incidence for this time for males and females in all four age groups (Supplementary Table S11).

### Incidence trends by ethnicity

After adjustment for age and sex, incidence trends differed by ethnicity (Table 1). Among individuals aged 20–29 years, incidence increased in all ethnic groups but rose most rapidly in Black individuals with a 4.1% increase per annum, almost double that observed in white individuals (RR 1.041 [1.030–1.052]) compared with White individuals (RR 1.022, [1.019–1.026]; interaction p = 0.002). The increase in Asian individuals was 2.8% per annum and not significantly different from White (RR 1.028, [1.021–1.034]; p = 0.118).

In those aged 30–39 years, all ethnic groups showed an increasing trend but occurred most rapidly in White individuals (RR 1.028 [1.026–1.030]), almost double the increase per annum in Asian and Black ethnic groups; Asian (RR 1.008, [1.005–1.010]) and Black individuals (RR 1.014, [1.009–1.019]; interaction p < 0.0001).

Incidence remained relatively stable across ethnicities with only modest rises in White and Black individuals aged 40–59 years; annual change in incidence was small at +0.4% per annum in White (RR 1.004, [1.003–1.004]) and Black groups (RR1.004, [1.002–1.006], p < 0.0001) but those from Asian ethnic groups showed no change over time (RR 1.000 [0.999–1.002], compared to White individuals p = 0.44).

In individuals aged 60–79 years, a modestly faster decline in White individuals than in Asian or Black individuals was observed; RR 0.988, [0.986–0.989], interaction p < 0.0001 for Asians and RR 0.986, [0.983–0.988] for Black, interaction p < 0.0001 compared to White (RR 0.980, [0.980–0.981]).

Examining more closely the ethnicity and sex trends in younger age groups (Table 1). In 20–29-year-olds, the increasing trend observed overall was primarily driven by increases in females (Table 1). Compared to White females, Asian and Black females demonstrated a faster rate of increase over time; White females 2.5% increase per year (RR 1.025 [1.021–1.030]), Black females a 4.5% increase per year (RR 1.045 [1.031–1.059]) and Asian females 4.4% per year (RR 1.044 [1.036–1.053]). In males, RRs were highest in Black males 3.5% per year (RR 1.035 [1.018–1.052]) but not significantly different from White males (RR 1.018 [1.012–1.024]).

In the 30–39-year-old age group, the overall rising trend was highest in White females (RR 1.038 [1.035–1.041]) with Asian females (RR 1.016 [1.012–1.020]) and Black females (RR 1.016 [1.010–1.023]) showing slightly lower increases per year. In males, however the fastest rise was observed in White (RR 1.018 [1.015–1.020]) and Black (RR 1.012 [1.005–1.018]), with no change in Asian males (RR 1.001 [0.998–1.005]).

### BMI at diagnosis

**Fig. 3:**
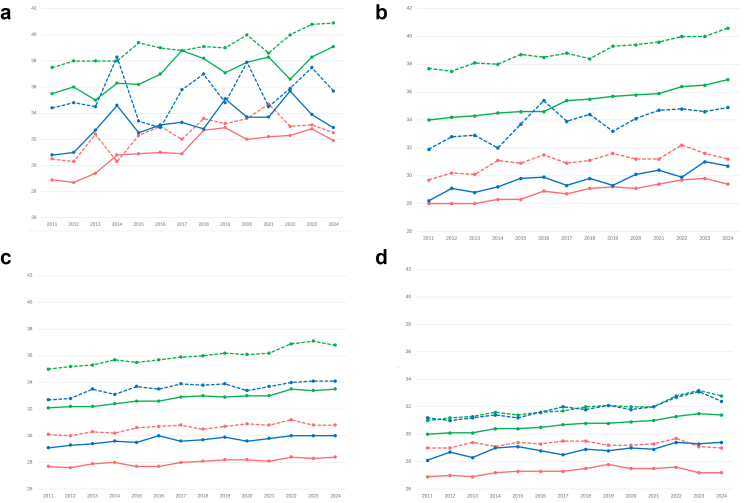
Median BMI within 26 weeks of diagnosis from 2011 to 2024 by age, sex and ethnicity. Panel a) individuals aged 20–29 years old, b) 30–39 years old, c) 40–59 years old and d) 60–79 years old. Dotted lines are females and solid lines are males. Green lines are white individuals; red lines are Asian individuals and blue lines are Black individuals

Median BMI within 26 weeks of diagnosis was higher in those aged 20-to-29-years and 30-to-39-years compared to those aged 60–79 years old (p < 0.005) (Fig. 3 and Supplementary Tables S12 and S13); for example in 2024 median BMI [IQR] in females aged 20–29 was 37.4 [31.2–44.6], 36.8 [30.9–43.8] in 30–39, 34.7 [30.0–40.5] in 40–59 and 32.1 [29.0–37.0] in 60–79 year-olds.

**Fig. 4:**
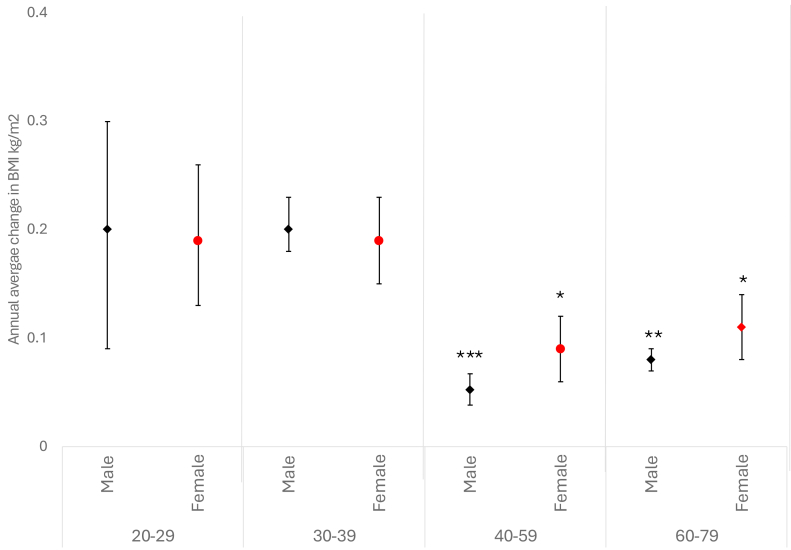
Average change with 95% confidence intervals in BMI (kg/m^2^) per annum by age and sex between 2011 and 2024. Statistical testing compared males and females in each age category to males and females in the 20–29 year old age group, respectively. ∗∗∗ = p < 0.001; ∗∗ = p < 0.01; ∗ = p < 0.05.

The annual increase in those with a recorded BMI was higher in females aged 20 to 29 (0.17 kg/m^2^ [95% CI 0.09–0.26]) and aged 30–39 years (0.20 kg/m^2^ [0.16–0.24]) than in those aged 40–59 years (0.09 kg/m^2^ per annum [0.06–0.12]) (Fig. 4). Both 40–59-year-old (p = 0.013) and 60–79 year old females (p = 0.045) had significantly lower rate of increase in BMI than 20–29 year olds.

Similarly, in males the annual increase in BMI varied by age with those in the younger age groups (20–29 years 0.17 kg/m^2^ [0.08–0.26], 30–39 years 0.21 kg/m^2^ [0.18–0.24]) demonstrating greater increases vs. 40–59 years olds (0.06 kg/m^2^ [0.04–0.07]) and 60–79 years (0.08 kg/m^2^ [0.06–0.09]) (Fig. 4). Both 40–59 year old (p < 0.001) and 60–79 year old males (p = 0.003) had significantly lower rate of increase in BMI than 20–29 year olds.

There were no statistically significant differences in the annual change in BMI over time by ethnicity (Supplementary Tables S14–S16), however in every age group, the median BMI was significantly lower in Asians vs. Black and White ethnicities and highest in the white group (p < 0.005).

## Discussion

### Summary of findings

Our findings advance understanding of type 2 diabetes epidemiology through a comprehensive, stratified population analysis, revealing striking shifts in incidence patterns across age, sex, and ethnicity. Over 14 years, incidence has risen sharply among younger adults, aged 20–29 years and 30–39 years (especially females), modestly in males and females aged 40–59 years, whilst declining in older age subgroups. Although increasing incidence was observed across ethnicity and sex sub-groups (except Asian males) in 20–29-year-olds, the rising incidence in 30–39-year-olds was driven largely by increases in White, Asian and Black females and White and Black males (in Asian males’ incidence were unchanged). Notably in older age groups, White females showed a rising trend while all other ethnicity and sex subgroups showing minimal change or a decline. Finally, while BMI at diagnosis of type 2 diabetes has steadily increased over time across age and sex sub-groups, the greatest rise, and highest median BMI, was observed in those aged 20–39 years (double the proportional increase per annum compared to older age groups). Together, these findings suggest that the drivers of type 2 diabetes are exerting a disproportionate impact on younger adults, emphasising the urgent need to tailor type 2 diabetes prevention strategies towards younger individuals.

### Trends in incidence

It is important to consider whether the diverging trends in incidence by age group represent a genuine transition of type 2 diabetes risk from older to younger age groups or whether they reflect other factors.

A reduction in incidence of type 2 diabetes in older individuals could be explained by type 2 diabetes prevention efforts. England, like other high-income countries^14^^,^^15^ implemented the National Diabetes Prevention Programme from 2016, directing a behavioural intervention to adults with non-diabetic hyperglycaemia and has shown to reduce type 2 diabetes incidence overall.^16^^,^^17^ However, the declining incidence in older age groups in our analysis pre-dates this intervention. Another explanation could be the increased awareness of type 2 diabetes risk through incentivised primary care initiatives^18^ that may have contributed to declining incidence in later life. The early years of the study period coincided with a national focus on case finding in those aged 40 years and older.^19^ These initiatives may have produced an initial rise in diagnosed incidence in older adults, followed by a later plateau as the pool of undiagnosed cases diminished and recorded incidence converged toward true incidence. The rise in median BMI at diagnosis has been smaller (compared to younger adults) in older adults, suggesting a stabilisation of this key modifiable risk factor.

A further contributory factor may be a cohort effect in which individuals with greater underlying susceptibility now develop diabetes earlier in life due to rising BMI and adverse lifestyle exposures. This earlier onset may deplete the pool of susceptible individuals who might otherwise have presented at older ages, contributing to declining incidence rates in later life, a pattern also observed in conditions such as colorectal cancer.^20^

In 2012 in England, HbA1c was instituted as a first line diagnostic test for diabetes easing barriers that may have precluded diagnostic testing.^21^ Some studies suggest that HbA1c detects fewer cases of diabetes than glucose tests.^22^ However, this would not adequately explain the rising incidence in younger individuals. In addition, younger individuals are not routinely screened for risk of type 2 diabetes as part of NHS Health Checks in England, beginning at 40-years.^19^

Potential miscoding of type 2 diabetes should be considered when interpreting trends. Misclassification of type 1 as type 2 diabetes is more likely at younger ages, where phenotypic overlap is greater, particularly among individuals with type 1 diabetes living with obesity.^23^^,^^24^ In sensitivity analyses, exclusion of adults coded with type 2 diabetes who initiated insulin within six months, did not materially alter trends. Early insulin use is also observed in early-onset type 2 diabetes and therefore does not confirm misclassification. The increasing clinical overlap highlights the need for careful phenotyping as previously recommended.^25^

### Sex differences

In our analysis the incidence of type 2 diabetes was higher in females than in males in those aged 20–29 years, but in all older age groups males had a higher incidence. Female preponderance in early-onset type 2 diabetes has been noted before in adolescent studies and other cross-sectional analyses.^1^^,^^2^^,^^7^^,^^26^ Our analysis shows that the female preponderance pattern does not extend across the whole ‘early-onset’ age range as 30- to 39-year-olds demonstrate male excess. This observed female excess may reflect that females are more susceptible due to greater weight gain.^26^^,^^27^ In the current analysis, and in other assessments of BMI at diagnosis of type 2 diabetes,^28^^,^^29^ median BMI is higher in females across all age groups, not just in those aged 20–29 years, so this seems unlikely to be the only factor.

The higher incidence in females may also reflect greater opportunistic testing for diabetes in females seeking healthcare for other reasons such as preconception, pregnancy or menstrual issues.^30^^,^^31^ The proportion of men with undiagnosed diabetes is likely to be higher as they are less likely to interact with healthcare services.^31^ Similarly, women developing gestational diabetes are also followed up for assessment of glycaemia post-partum and may be referred into the diabetes prevention programme.^32^ Of note increasing incidence was also observed in White females aged 40–59-year-olds, suggesting that prevention efforts may be less effective or that this group may have been at risk due to previous gestational diabetes, a known risk factor for later type 2 diabetes.

### Changes in BMI

BMI shortly after diagnosis of type 2 diabetes increased most notably in the youngest age groups. In all age groups, White males and females had the highest median BMI at diagnosis, consistent with the observation that non-White ethnicities develop type 2 diabetes at a lower BMI.^33^

While global obesity prevalence has risen,^34^ trends in BMI at diagnosis of type 2 diabetes (by age group) are understudied.^29^ Our analysis replicated known ethnic differences in BMI with Asian patients exhibiting significantly lower BMI at every time point and age group.^33^^,^^35^ However, in younger age groups the BMI in Asian individuals was still high confirming the necessity of significant obesity to develop early-onset type 2 diabetes.

Early-onset of type 2 diabetes demonstrates a greater deviation of metabolic risk factors from ‘healthy’ compared to later-onset type 2 diabetes^36^; the greater increase in BMI among younger adults suggests that successive cohorts are developing type 2 diabetes at higher levels of obesity driving earlier diagnosis. Earlier onset linked to severe obesity may, in turn, account for the smaller BMI increases observed in older groups, since susceptible individuals have developed type 2 diabetes earlier. BMI was lower in older individuals, while still exceeding ethnicity-adjusted obesity thresholds.^35^ As ageing is associated with progressive beta-cell dysfunction and increased diabetes susceptibility, these data support that the degree of excess adiposity required to trigger type 2 diabetes in later life, is lower.^29^

### Findings in relation to other studies

Our findings are similar to estimates from the Global Burden of Disease study, which reported an increase incidence estimates in most high-income countries and estimated a 2019 incidence in the UK of 346.95 per 100,000 person years in people aged 15–39 years.^2^ The incidence in our study is stratified by age and sex category but is lower (e.g., in females aged 30–39 years, incidence was 198 per 100,000 person-years). This may reflect that the Global Burden of Disease study includes estimates of undiagnosed diabetes, unlike NDA data which is based on diagnosed cases.

The GLOBODIAB consortium estimated incidence of type 2 diabetes in 15- to 39-year-olds across 8 high-income countries and showed variable trends with increasing incidence in Denmark and Finland, a reducing incidence in Spain and Scotland and unchanged in Hungary.^9^ The declining trend in Scotland, contrasting our analysis in England, may reflect that data collection for Scotland ended in 2020 when incidence was lower than previous years due to the COVID-19 pandemic affecting use of non-emergency healthcare. In contrast this analysis includes increases in incidence post-pandemic. However, in England incidence of type 2 diabetes in younger adults was rising prior to the pandemic. Another pre-pandemic analysis from Sweden, also showed increasing incidence in young adults.^8^

A systematic review of 47 studies from predominantly European populations, showed two-thirds of global regions observed a decrease or plateauing of incidence in recent years.^37^ Thus, our observed decline in older populations, which proportionally represents most incident type 2 diabetes cases, is in keeping with other analyses.

### Impact of findings

The rising incidence of type 2 diabetes among younger adults has major implications. Early-onset disease progresses more aggressively,^38^^,^^39^ carries higher complication rates,^4^^,^^6^ and creates a substantial long-term healthcare burden.^37^^,^^38^ Although absolute numbers remain lower than in older adults, the expected morbidity in this younger cohort is concerning.^40^ The extremely high BMI in early-onset cases highlights the need for tailored, weight-focused treatment approaches to mitigate lifetime complication risk. Type 2 diabetes remission should also be proactively considered as early as possible.

These findings highlight the urgent need for targeted prevention strategies. Rising BMI in younger adults indicates that existing obesity prevention efforts are inadequate. Interventions must prioritise healthy diet, physical activity and the socioeconomic determinants of early-onset severe obesity, alongside improved risk-based screening. Programmes such as the National Diabetes Prevention Programme have achieved reasonable uptake among younger adults through digital delivery but wider participation is needed.^41^ Population studies show a higher prevalence of undiagnosed diabetes in younger adults,^42^^,^^43^ suggesting that prediabetes-based recruitment misses at-risk younger individuals.

### Strengths and limitations

The strength of our analysis is that is uses data from a health service with universal coverage, free at the point of care and with incentivised annual data collection. However, there are some limitations. NDA coverage varied over the study period, and we assumed no systematic differences between participating and non-participating GP practices. As participation was lower in earlier years, this assumption may have led to under- or overestimation of trends. Second, the dataset only includes diagnosed cases of type 2 diabetes and does not account for undiagnosed individuals, underestimating the true burden of disease. Third, type of diabetes is defined using diagnostic codes, however our sensitivity analysis provides reassurance that changes in trends observed are not accounted for by potential rises in miscoding of type 2 diabetes in young people. Fourth we studied BMI within 26-weeks of diagnosis and acknowledge that BMI at diagnosis may be different. Fifth, we reported the incidence of diagnosed type 2 diabetes of at least 18-months duration, which has the potential to introduce selection bias by excluding individuals who died or de-registered prior to data extraction. Our sensitivity analysis shows that this delay did not alter the trends in incidence in the latter years of this analysis, when data extraction was contemporaneous. Sixth, due to the absence of annual ethnicity stratified population estimates, we used interpolation between two census timepoints to estimate ethnicity population denominators. Finally, as the NDA includes individuals with diagnosed diabetes, aggregate data were combined with national population estimates; exploration of trends by age and ethnicity was therefore limited to stratified analyses.

### Conclusion

This analysis highlights important shifts in the incidence of type 2 diabetes in England, with rising incidence in younger adults, particularly in females and across all ethnicities, contrasting declining trends at older ages. Whilst the overall incidence remains low in young adults these trends are a major concern due to the high risk of multiple long-term conditions and associated earlier mortality in affected individuals. The markedly increasing BMI at diagnosis among younger adults, suggests that this group may be uniquely susceptible to environmental factors driving obesity. Tailored, proactive screening and interventions that address these distinct susceptibilities are essential to reverse these trends. By learning from the changes observed in older populations and tailoring prevention approaches to younger adults, there is significant potential to mitigate the rising burden of early-onset type 2 diabetes.

## Contributors

SM & BY conceived the study. NH & JO had full access to the data, verified the data analysis and carried out the formal statistical analysis.

All authors collaborated in interpretation of the results and drafting of the manuscript. All authors contributed to the decision to submit the manuscript.

## Data sharing statement

The National Diabetes Audit data can be requested via the NHS England Data Access Request Service.

## Declaration of interests

NH has received payments from AstraZeneca for participation in clinical trials. KK has received grant funding from AstraZeneca, Boehringer Ingelheim, Lilly, MSD, Novo Nordisk, Sanofi, Servier, Oramed Pharmaceuticals, Roche, Daiichi-Sankyo, Applied Therapeutics and declares consulting fees/honoraria from Amgen, AstraZeneca, Bristol Myers Squibb, Boehringer Ingelheim, Lilly, Novo Nordisk, Sanofi, Servier, Pfizer, Roche, Daiichi-Sankyo, Embecta and Nestle Health Science. JV is the National Specialty Advisor for Multiple Long-Term Conditions at NHS England, was the National Clinical Director for Diabetes and Obesity at NHS England from April 2013 to September 2023 and was an International Advisor for the Ministry of Public Health in Qatar’s “Diabetes, Obesity and Atherosclerotic Cardiovascular Disease Strategy 2024 to 2030”, for a 3-month period in 2024. CH is member of the clinical committee of the RECORD study which is sponsored by Abbott. SM has received honoraria for educational presentations from Sanofi, Menarini and Lilly and participated in an advisory board for Insulet. All other authors have no disclosures.
