# Supplementary material for: Trends in type 2 diabetes incidence and BMI at diagnosis by age, sex, and ethnicity, 2011–2024: a population-based analysis of the English National Diabetes Audit
Source: Lancet Reg Health Eur. 2026 Jul 22;67:101764. doi: 10.1016/j.lanepe.2026.101764 (PMC13453952; doi:10.1016/j.lanepe.2026.101764)
Supplement: Supplementary file 1 [file mmc1.docx]

**Supplementary Tables and Figures**

Supplementary figure S1: Diagram depicting the rationale for calculating incidence of 18 months duration across the entire study period and sensitivity analaysis conducted in latter audit years

Supplementary Table S1: Number of incident cases of type 2 diabetes identified for each year by age and sex

|  | | Number identified in NDA data collection | | | | | | | |
| --- | --- | --- | --- | --- | --- | --- | --- | --- | --- |
|  |  | Males | | | | Female | | | |
|  |  | 20-29 | 30-39 | 40-59 | 60-79 | 20-29 | 30-39 | 40-59 | 60-79 |
| Incident | 2011 | 570 | 4,050 | 34,260 | 41,120 | 720 | 3,115 | 21,650 | 31,495 |
|  | 2012 | 670 | 4,245 | 36,820 | 43,395 | 870 | 3,260 | 23,170 | 32,480 |
|  | 2013 | 605 | 4,490 | 37,745 | 42,450 | 875 | 3,475 | 24,485 | 32,510 |
|  | 2014 | 555 | 3,825 | 31,845 | 35,450 | 735 | 3,085 | 21,120 | 27,470 |
|  | 2015 | 435 | 2,960 | 25,125 | 28,725 | 615 | 2,580 | 17,285 | 23,320 |
|  | 2016 | 412 | 2,925 | 24,165 | 25,985 | 675 | 2,470 | 16,580 | 20,700 |
|  | 2017 | 690 | 4,640 | 38,595 | 40,055 | 980 | 4,010 | 26,395 | 32,310 |
|  | 2018 | 850 | 5,500 | 45,025 | 46,115 | 1,180 | 4,635 | 31,145 | 36,470 |
|  | 2019 | 860 | 5,560 | 44,155 | 43,930 | 1,235 | 4,890 | 30,245 | 33,760 |
|  | 2020 | 920 | 5,740 | 46,525 | 49,460 | 1,280 | 5,125 | 32,815 | 37,910 |
|  | 2021 | 880 | 5,805 | 48,190 | 49,806 | 1,380 | 5,670 | 34,575 | 38,850 |
|  | 2022 | 775 | 5,130 | 38,660 | 38,870 | 1,320 | 5,120 | 28,405 | 29,960 |
|  | 2023 | 1,135 | 6,845 | 51,100 | 52,335 | 1,705 | 6,990 | 40,105 | 41,550 |
|  | 2024 | 1,130 | 7,510 | 55,440 | 58,640 | 1,780 | 7,145 | 41,765 | 45,765 |

The year displayed indicates the year of the data extraction

Supplementary Table S2: Number of incident cases of type 2 diabetes adjusted to reflect coverage of the NDA for each year by age and sex

|  | | Number adjusted to reflect coverage of NDA | | | | | | | |
| --- | --- | --- | --- | --- | --- | --- | --- | --- | --- |
|  |  | Males | | | | Females | | | |
|  |  | 20-29 years | 30-39 years | 40-59 years | 60-79 years | 20-29 years | 30-39 years | 40-59  years | 60-79 years |
| Incident | 2011 | 725 | 5,140 | 43,475 | 52,180 | 910 | 3,955 | 27,470 | 39,965 |
|  | 2012 | 810 | 5,125 | 44,440 | 52,375 | 1,050 | 3,930 | 27,960 | 39,200 |
|  | 2013 | 745 | 5,540 | 46,560 | 52,360 | 1,080 | 4,290 | 30,200 | 40,100 |
|  | 2014 | 785 | 5,405 | 44,980 | 50,070 | 1,035 | 4,360 | 29,845 | 38,805 |
|  | 2015 | 790 | 5,390 | 45,740 | 52,295 | 1,120 | 4,695 | 31,465 | 42,470 |
|  | 2016 | 750 | 5,325 | 43,980 | 47,295 | 1,230 | 4,495 | 30,170 | 37,675 |
|  | 2017 | 850 | 5,705 | 47,435 | 49,230 | 1,205 | 4,930 | 32,440 | 39,710 |
|  | 2018 | 865 | 5,545 | 45,380 | 46,480 | 1,190 | 4,670 | 31,390 | 36,755 |
|  | 2019 | 875 | 5,660 | 44,940 | 44,715 | 1,255 | 4,980 | 30,785 | 34,360 |
|  | 2020 | 935 | 5,865 | 47,530 | 50,525 | 1,310 | 5,240 | 33,525 | 38,725 |
|  | 2021 | 890 | 5,850 | 48,570 | 50,200 | 1,390 | 5,705 | 34,850 | 39,155 |
|  | 2022 | 780 | 5,170 | 38,960 | 39,175 | 1,330 | 5,160 | 28,630 | 30,195 |
|  | 2023 | 1,150 | 6,960 | 51,975 | 53,230 | 1,735 | 7,110 | 40,790 | 42,260 |
|  | 2024 | 1,150 | 7,645 | 56,440 | 59,695 | 1,810 | 7,275 | 42,515 | 46,590 |

Supplementary Table S3: Crude incidence of type 2 diabetes per 1000 by age and sex

|  | | Crude prevalence per 1000 | | | | | | | |
| --- | --- | --- | --- | --- | --- | --- | --- | --- | --- |
|  |  | Males | | | | Females | | | |
|  |  | 20-29 years | 30-39 years | 40-59 years | 60-79 years | 20-29 years | 30-39 years | 40-59 years | 60-79 years |
| Incident | 2011 | 0.21 | 1.5 | 6.1 | 11.5 | 0.26 | 1.1 | 3.8 | 8.1 |
|  | 2012 | 0.23 | 1.5 | 6.2 | 11.4 | 0.30 | 1.1 | 3.9 | 7.9 |
|  | 2013 | 0.21 | 1.6 | 6.5 | 11.3 | 0.30 | 1.2 | 4.1 | 8.0 |
|  | 2014 | 0.22 | 1.5 | 6.3 | 10.6 | 0.29 | 1.2 | 4.0 | 7.6 |
|  | 2015 | 0.22 | 1.5 | 6.3 | 10.9 | 0.30 | 1.3 | 4.2 | 8.2 |
|  | 2016 | 0.21 | 1.5 | 6.0 | 9.8 | 0.33 | 1.2 | 4.0 | 7.2 |
|  | 2017 | 0.23 | 1.5 | 6.5 | 10.0 | 0.32 | 1.3 | 4.3 | 7.5 |
|  | 2018 | 0.23 | 1.5 | 6.2 | 9.3 | 0.31 | 1.2 | 4.2 | 6.8 |
|  | 2019 | 0.23 | 1.5 | 6.1 | 8.8 | 0.32 | 1.3 | 4.1 | 6.3 |
|  | 2020 | 0.25 | 1.6 | 6.5 | 9.8 | 0.33 | 1.3 | 4.4 | 7.0 |
|  | 2021 | 0.24 | 1.6 | 6.7 | 9.5 | 0.35 | 1.4 | 4.6 | 6.9 |
|  | 2022 | 0.21 | 1.4 | 5.3 | 7.3 | 0.33 | 1.3 | 3.8 | 5.2 |
|  | 2023 | 0.30 | 1.8 | 6.9 | 9.8 | 0.42 | 1.7 | 5.4 | 7.2 |
|  | 2024 | 0.30 | 2.0 | 7.6 | 10.8 | 0.44 | 1.8 | 5.6 | 7.8 |

Supplementary Table S4: Directly age standardised incidence of type 2 diabetes per 1000 by age and sex, 2011-2024

|  | 20-29 years | | 30-39 years | | 40-59 years | | 60-79 years | |
| --- | --- | --- | --- | --- | --- | --- | --- | --- |
|  | Male | Female | Male | Female | Male | Female | Male | Female |
| 2011 | 0.21 (0.19-0.22) | 0.26 (0.24-0.27) | 1.46 (1.42-1.5) | 1.12 (1.08-1.15) | 6.1 (5.9-6.4) | 4 (3.9-4.2) | 11.6 (11.2-12.1) | 8.1 (7.8-8.5) |
| 2012 | 0.23 (0.22-0.25) | 0.3 (0.28-0.32) | 1.47 (1.43-1.51) | 1.12 (1.08-1.15) | 6.3 (6.1-6.5) | 4 (3.9-4.2) | 11.4 (11.1-12) | 7.9 (7.6-8.3) |
| 2013 | 0.22 (0.2-0.23) | 0.31 (0.29-0.33) | 1.59 (1.55-1.63) | 1.21 (1.18-1.25) | 6.6 (6.4-6.8) | 4.3 (4.1-4.5) | 11.2 (10.9-11.8) | 7.9 (7.6-8.4) |
| 2014 | 0.22 (0.21-0.24) | 0.29 (0.27-0.31) | 1.54 (1.5-1.58) | 1.22 (1.18-1.26) | 6.3 (6.1-6.6) | 4.2 (4-4.4) | 10.6 (10.2-11.1) | 7.6 (7.3-8) |
| 2015 | 0.22 (0.21-0.24) | 0.31 (0.29-0.33) | 1.51 (1.47-1.55) | 1.29 (1.25-1.33) | 6.4 (6.2-6.6) | 4.3 (4.2-4.5) | 10.9 (10.6-11.5) | 8.2 (7.9-8.6) |
| 2016 | 0.21 (0.19-0.22) | 0.33 (0.31-0.35) | 1.47 (1.43-1.51) | 1.21 (1.17-1.24) | 6 (5.9-6.3) | 4.1 (4-4.3) | 9.7 (9.4-10.3) | 7.2 (6.9-7.6) |
| 2017 | 0.23 (0.22-0.25) | 0.32 (0.3-0.34) | 1.55 (1.51-1.59) | 1.3 (1.26-1.33) | 6.5 (6.3-6.8) | 4.4 (4.2-4.6) | 10 (9.7-10.5) | 7.4 (7.2-7.9) |
| 2018 | 0.23 (0.22-0.25) | 0.31 (0.29-0.33) | 1.49 (1.45-1.53) | 1.2 (1.17-1.24) | 6.2 (6-6.4) | 4.2 (4-4.4) | 9.3 (9-9.8) | 6.8 (6.5-7.2) |
| 2019 | 0.23 (0.22-0.25) | 0.32 (0.3-0.34) | 1.51 (1.47-1.55) | 1.27 (1.23-1.3) | 6.1 (5.9-6.4) | 4.1 (3.9-4.3) | 8.8 (8.5-9.3) | 6.3 (6-6.7) |
| 2020 | 0.25 (0.24-0.27) | 0.33 (0.31-0.35) | 1.57 (1.53-1.61) | 1.32 (1.29-1.36) | 6.5 (6.3-6.8) | 4.4 (4.3-4.7) | 9.8 (9.5-10.3) | 6.9 (6.7-7.4) |
| 2021 | 0.24 (0.22-0.25) | 0.35 (0.33-0.37) | 1.56 (1.52-1.61) | 1.43 (1.39-1.47) | 6.7 (6.5-6.9) | 4.6 (4.4-4.8) | 9.6 (9.2-10.1) | 6.9 (6.6-7.3) |
| 2022 | 0.21 (0.19-0.22) | 0.33 (0.31-0.35) | 1.37 (1.33-1.4) | 1.28 (1.24-1.31) | 5.3 (5.1-5.5) | 3.8 (3.6-4) | 7.3 (7.1-7.8) | 5.2 (5-5.6) |
| 2023 | 0.3 (0.28-0.32) | 0.42 (0.4-0.44) | 1.81 (1.76-1.85) | 1.73 (1.69-1.77) | 6.9 (6.7-7.2) | 5.4 (5.2-5.6) | 9.8 (9.5-10.3) | 7.2 (6.9-7.6) |
| 2024 | 0.3 (0.28-0.32) | 0.44 (0.42-0.46) | 1.98 (1.94-2.03) | 1.76 (1.72-1.8) | 7.5 (7.3-7.9) | 5.6 (5.4-5.8) | 10.8 (10.5-11.4) | 7.8 (7.5-8.2) |

Supplementary Table S5: Directly age standardised incidence of type 2 diabetes per 1000 by age, sex and ethnicity, 2011-2024

|  | | Asian | | Black | | White | |
| --- | --- | --- | --- | --- | --- | --- | --- |
|  |  | Male | Female | Male | Female | Male | Female |
| 20-29 years | 2011 | 0.51 (0.44-0.58) | 0.47 (0.41-0.54) | 0.41 (0.31-0.53) | 0.38 (0.29-0.49) | 0.14 (0.12-0.15) | 0.20 (0.18-0.22) |
|  | 2012 | 0.48 (0.41-0.55) | 0.50 (0.43-0.57) | 0.36 (0.27-0.48) | 0.45 (0.35-0.56) | 0.17 (0.16-0.19) | 0.24 (0.22-0.26) |
|  | 2013 | 0.48 (0.41-0.55) | 0.54 (0.47-0.61) | 0.35 (0.26-0.46) | 0.55 (0.44-0.67) | 0.15 (0.14-0.17) | 0.24 (0.22-0.26) |
|  | 2014 | 0.47 (0.41-0.55) | 0.52 (0.46-0.60) | 0.45 (0.35-0.58) | 0.48 (0.38-0.6) | 0.16 (0.14-0.17) | 0.23 (0.22-0.25) |
|  | 2015 | 0.49 (0.43-0.56) | 0.65 (0.57-0.73) | 0.52 (0.41-0.65) | 0.45 (0.35-0.56) | 0.15 (0.14-0.17) | 0.24 (0.22-0.26) |
|  | 2016 | 0.43 (0.37-0.49) | 0.60 (0.53-0.68) | 0.34 (0.25-0.45) | 0.51 (0.41-0.63) | 0.16 (0.14-0.17) | 0.27 (0.25-0.29) |
|  | 2017 | 0.45 (0.39-0.52) | 0.54 (0.48-0.62) | 0.43 (0.33-0.56) | 0.56 (0.45-0.68) | 0.17 (0.16-0.19) | 0.26 (0.24-0.28) |
|  | 2018 | 0.49 (0.42-0.56) | 0.54 (0.48-0.62) | 0.29 (0.21-0.39) | 0.44 (0.35-0.55) | 0.17 (0.15-0.18) | 0.25 (0.24-0.27) |
|  | 2019 | 0.39 (0.33-0.45) | 0.63 (0.56-0.71) | 0.39 (0.30-0.50) | 0.50 (0.40-0.61) | 0.18 (0.16-0.19) | 0.25 (0.23-0.27) |
|  | 2020 | 0.47 (0.41-0.54) | 0.60 (0.53-0.68) | 0.52 (0.41-0.65) | 0.62 (0.51-0.74) | 0.18 (0.17-0.20) | 0.27 (0.25-0.28) |
|  | 2021 | 0.44 (0.38-0.51) | 0.76 (0.68-0.85) | 0.44 (0.34-0.56) | 0.52 (0.42-0.63) | 0.17 (0.16-0.19) | 0.26 (0.25-0.28) |
|  | 2022 | 0.37 (0.32-0.43) | 0.56 (0.49-0.63) | 0.47 (0.36-0.59) | 0.60 (0.50-0.73) | 0.15 (0.13-0.16) | 0.26 (0.25-0.28) |
|  | 2023 | 0.56 (0.50-0.63) | 0.80 (0.72-0.89) | 0.69 (0.57-0.84) | 0.90 (0.77-1.05) | 0.20 (0.19-0.22) | 0.32 (0.30-0.34) |
|  | 2024 | 0.63 (0.56-0.70) | 1.02 (0.93-1.12) | 0.59 (0.47-0.72) | 0.70 (0.59-0.83) | 0.20 (0.18-0.21) | 0.32 (0.30-0.34) |
| 30-39 years | 2011 | 3.46 (3.28-3.65) | 2.47 (2.31-2.63) | 2.79 (2.52-3.07) | 2.08 (1.86-2.31) | 0.96 (0.92-0.99) | 0.79 (0.75-0.82) |
|  | 2012 | 3.76 (3.58-3.96) | 2.55 (2.39-2.72) | 2.59 (2.33-2.87) | 2.03 (1.82-2.27) | 0.92 (0.88-0.95) | 0.79 (0.76-0.82) |
|  | 2013 | 4.27 (4.07-4.48) | 2.83 (2.67-3.01) | 2.69 (2.43-2.98) | 2.31 (2.08-2.55) | 0.98 (0.94-1.02) | 0.83 (0.79-0.86) |
|  | 2014 | 4.13 (3.94-4.34) | 2.87 (2.70-3.04) | 2.52 (2.27-2.80) | 2.50 (2.26-2.75) | 0.94 (0.90-0.97) | 0.81 (0.78-0.85) |
|  | 2015 | 4.27 (4.08-4.48) | 3.31 (3.13-3.49) | 2.78 (2.51-3.06) | 2.49 (2.25-2.74) | 0.90 (0.86-0.93) | 0.85 (0.81-0.88) |
|  | 2016 | 4.02 (3.83-4.22) | 3.11 (2.94-3.28) | 2.89 (2.62-3.18) | 2.21 (2.00-2.45) | 0.87 (0.84-0.91) | 0.79 (0.76-0.83) |
|  | 2017 | 4.21 (4.02-4.41) | 3.38 (3.21-3.56) | 2.83 (2.56-3.11) | 2.27 (2.05-2.50) | 0.93 (0.89-0.96) | 0.87 (0.84-0.90) |
|  | 2018 | 3.79 (3.61-3.98) | 2.87 (2.72-3.04) | 2.73 (2.47-3.01) | 2.07 (1.86-2.29) | 0.92 (0.89-0.96) | 0.83 (0.80-0.87) |
|  | 2019 | 3.83 (3.65-4.01) | 3.00 (2.84-3.16) | 2.54 (2.29-2.81) | 2.01 (1.81-2.23) | 0.95 (0.92-0.99) | 0.89 (0.85-0.92) |
|  | 2020 | 4.02 (3.83-4.20) | 3.16 (3.00-3.33) | 2.59 (2.34-2.86) | 2.24 (2.03-2.47) | 0.99 (0.95-1.03) | 0.91 (0.88-0.95) |
|  | 2021 | 3.99 (3.81-4.18) | 2.99 (2.85-3.15) | 2.55 (2.30-2.82) | 2.37 (2.15-2.60) | 0.98 (0.95-1.02) | 0.99 (0.96-1.03) |
|  | 2022 | 2.93 (2.78-3.09) | 2.59 (2.45-2.74) | 2.60 (2.35-2.87) | 2.07 (1.86-2.28) | 0.92 (0.88-0.95) | 0.96 (0.93-1.00) |
|  | 2023 | 3.89 (3.71-4.07) | 3.42 (3.26-3.59) | 3.26 (2.98-3.56) | 2.94 (2.70-3.19) | 1.23 (1.19-1.27) | 1.31 (1.27-1.35) |
|  | 2024 | 4.77 (4.58-4.97) | 3.65 (3.48-3.82) | 3.51 (3.22-3.83) | 2.99 (2.75-3.24) | 1.25 (1.21-1.30) | 1.30 (1.26-1.34) |
| 40-59 years | 2011 | 13.3 (12.4-14.7) | 10.1 (9.3-11.2) | 11.6 (10.6-13.2) | 7.9 (7.1-9.1) | 5.4 (5.3-5.6) | 3.2 (3.1-3.4) |
|  | 2012 | 12.6 (11.8-13.9) | 9.0 (8.4-10.1) | 11.7 (10.7-13.4) | 8.2 (7.5-9.4) | 5.4 (5.3-5.7) | 3.2 (3.1-3.4) |
|  | 2013 | 12.9 (12.1-14.3) | 9.5 (8.8-10.6) | 12.1 (11.1-13.8) | 9.2 (8.4-10.5) | 5.6 (5.4-5.8) | 3.4 (3.3-3.6) |
|  | 2014 | 12.5 (11.6-13.8) | 9.6 (8.9-10.6) | 12.0 (11.0-13.7) | 8.8 (8.1-10.0) | 5.3 (5.1-5.5) | 3.3 (3.2-3.5) |
|  | 2015 | 12.6 (11.8-14.0) | 9.6 (8.9-10.6) | 12.3 (11.2-14) | 9.9 (9.1-11.2) | 5.2 (5.0-5.4) | 3.4 (3.2-3.6) |
|  | 2016 | 11.9 (11.1-13.2) | 8.5 (7.9-9.5) | 11.6 (10.5-13.4) | 9.2 (8.4-10.5) | 4.9 (4.7-5.1) | 3.2 (3.1-3.4) |
|  | 2017 | 12.7 (11.8-14.0) | 9.4 (8.7-10.4) | 13.0 (11.8-14.9) | 10.2 (9.2-11.6) | 5.2 (5.0-5.4) | 3.3 (3.2-3.6) |
|  | 2018 | 11.7 (10.9-13.0) | 8.6 (7.9-9.5) | 11.6 (10.5-13.4) | 9.1 (8.2-10.5) | 4.9 (4.7-5.2) | 3.2 (3.1-3.4) |
|  | 2019 | 11.6 (10.8-12.9) | 8.1 (7.5-9.1) | 11.2 (10.1-13.0) | 8.6 (7.7-10.0) | 4.8 (4.6-5.0) | 3.1 (3.0-3.4) |
|  | 2020 | 12.0 (11.2-13.2) | 8.8 (8.2-9.8) | 11.6 (10.5-13.4) | 9.0 (8.1-10.2) | 5.1 (4.9-5.3) | 3.4 (3.3-3.6) |
|  | 2021 | 12.8 (12.1-14.0) | 9.4 (8.8-10.3) | 11.7 (10.5-13.5) | 9.0 (8.2-10.4) | 5.1 (4.9-5.4) | 3.5 (3.3-3.7) |
|  | 2022 | 9.4 (8.8-10.3) | 6.6 (6.2-7.4) | 9.6 (8.6-11.2) | 7.5 (6.7-8.7) | 4.2 (4.0-4.4) | 3.0 (2.8-3.2) |
|  | 2023 | 13.1 (12.4-14.1) | 10.2 (9.6-11) | 12 (10.9-13.8) | 9.8 (9.0-11.1) | 5.5 (5.3-5.8) | 4.2 (4.0-4.4) |
|  | 2024 | 14.5 (13.8-15.6) | 10.7 (10.2-11.5) | 13.1 (11.8-14.9) | 9.8 (8.9-11.1) | 5.9 (5.7-6.2) | 4.3 (4.2-4.6) |
| 60-79 years | 2011 | 15.8 (13.5-19.5) | 13.1 (11.2-16.1) | 20.8 (15.9-28.6) | 18.1 (14.0-24.5) | 10.9 (10.5-11.4) | 7.5 (7.2-7.9) |
|  | 2012 | 15.0 (12.6-18.9) | 13.3 (11.3-16.5) | 19.2 (14.7-26.5) | 16.6 (12.9-22.5) | 10.8 (10.4-11.3) | 7.1 (6.9-7.6) |
|  | 2013 | 15.6 (13.1-19.6) | 14.2 (12.1-17.5) | 20.9 (16.7-27.7) | 17.8 (14.3-23.5) | 10.5 (10.1-11.1) | 7.1 (6.8-7.5) |
|  | 2014 | 15.3 (12.8-19.1) | 14.2 (12.1-17.5) | 19.4 (15.6-25.6) | 18.1 (14.6-23.5) | 9.8 (9.5-10.4) | 6.7 (6.5-7.2) |
|  | 2015 | 16.6 (14.1-20.6) | 16.1 (13.9-19.5) | 21.2 (17.3-27.5) | 19.5 (16.2-24.7) | 10.1 (9.8-10.7) | 7.2 (6.9-7.7) |
|  | 2016 | 14.6 (12.4-18.2) | 13.7 (11.7-16.8) | 18.7 (15.4-24.0) | 18.5 (15.3-23.4) | 9.0 (8.6-9.5) | 6.3 (6.0-6.7) |
|  | 2017 | 15.9 (13.6-19.4) | 14.7 (12.7-17.7) | 18.5 (15.3-23.6) | 17.5 (14.6-22.2) | 9.2 (8.9-9.7) | 6.5 (6.3-7.0) |
|  | 2018 | 13.9 (11.9-17.0) | 12.9 (11.1-15.7) | 17.0 (14.0-21.9) | 16.6 (14.0-20.8) | 8.6 (8.2-9.1) | 5.9 (5.7-6.3) |
|  | 2019 | 13.3 (11.4-16.2) | 11.5 (9.9-14.0) | 15.3 (12.6-19.4) | 13.5 (11.2-17.3) | 8.1 (7.8-8.6) | 5.5 (5.2-5.9) |
|  | 2020 | 13.6 (11.9-16.4) | 12.4 (10.8-14.9) | 16.8 (14.2-20.8) | 14.7 (12.4-18.5) | 9.1 (8.7-9.6) | 6.1 (5.8-6.5) |
|  | 2021 | 14.7 (12.9-17.4) | 12.6 (11.1-15.1) | 16.1 (13.7-19.8) | 14.4 (12.3-17.7) | 8.8 (8.4-9.3) | 6.0 (5.7-6.4) |
|  | 2022 | 10.1 (8.7-12.2) | 8.6 (7.4-10.5) | 14.1 (11.9-17.5) | 10.7 (9.0-13.4) | 6.8 (6.5-7.2) | 4.6 (4.4-5.0) |
|  | 2023 | 15.5 (14.0-17.9) | 13.5 (12.0-15.8) | 21.2 (18.9-24.8) | 17.3 (15.2-20.6) | 8.9 (8.6-9.4) | 6.2 (5.9-6.6) |
|  | 2024 | 16.2 (14.7-18.5) | 13.4 (12.0-15.6) | 22.5 (20.3-25.9) | 18.5 (16.5-21.5) | 9.9 (9.5-10.4) | 6.8 (6.5-7.2) |

Supplementary table S6: Median age at diagnosis by age group, sex and year

|  | 20-29 years | | 30-39 years | | 40-59 years | | 60-79 years | |
| --- | --- | --- | --- | --- | --- | --- | --- | --- |
|  | Females | Males | Females | Males | Females | Males | Females | Males |
| 2011 | 26.9 (24.3-28.6) | 27.7 (25.5-29.0) | 36.4 (33.8-38.4) | 36.8 (34.2-38.6) | 52.3 (47.2-56.2) | 52.0 (47.2-56.1) | 69.4 (64.7-74.4) | 68.1 (64.1-73.2) |
| 2012 | 26.8 (24.4-28.6) | 27.4 (25.0-28.9) | 36.2 (33.6-38.3) | 36.8 (34.3-38.5) | 52.2 (47.2-56.2) | 52.0 (47.3-56.1) | 69.2 (64.8-74.2) | 68.1 (64.2-73.1) |
| 2013 | 27.1 (24.8-28.7) | 27.5 (25.5-29.0) | 36.3 (33.6-38.4) | 36.5 (33.9-38.4) | 52.2 (47.3-56.2) | 51.9 (47.2-56.1) | 69.1 (64.8-74.3) | 68.1 (64.3-73.0) |
| 2014 | 27.1 (24.6-28.7) | 27.4 (24.9-28.9) | 36.2 (33.8-38.3) | 36.7 (34.3-38.4) | 52.2 (47.4-56.2) | 52.0 (47.2-56.1) | 69.1 (64.8-74.2) | 68.2 (64.3-73.1) |
| 2015 | 26.7 (24.6-28.6) | 27.6 (25.4-29.1) | 36.2 (33.9-38.2) | 36.6 (34.3-38.4) | 52.4 (47.4-56.3) | 52.1 (47.3-56.1) | 69.2 (64.9-74.4) | 68.5 (64.4-73.3) |
| 2016 | 27.0 (24.3-28.7) | 27.3 (24.6-28.9) | 36.3 (33.9-38.2) | 36.4 (34.1-38.3) | 52.4 (47.5-56.2) | 52.1 (47.4-56.1) | 69.3 (64.7-74.2) | 68.7 (64.4-73.2) |
| 2017 | 27.0 (24.6-28.8) | 27.2 (24.9-28.9) | 36.3 (33.8-38.3) | 36.8 (34.3-38.5) | 52.4 (47.5-56.2) | 52.1 (47.3-56.1) | 69.4 (64.7-74.1) | 68.6 (64.2-73.2) |
| 2018 | 26.7 (24.1-28.5) | 27.3 (24.8-28.9) | 36.3 (33.6-38.3) | 36.7 (34.2-38.5) | 52.5 (47.7-56.3) | 52.2 (47.5-56.2) | 69.3 (64.5-74.1) | 68.5 (64.1-73.2) |
| 2019 | 26.8 (24.4-28.6) | 27.5 (24.9-28.9) | 36.2 (33.6-38.3) | 36.7 (34.2-38.6) | 52.4 (47.6-56.3) | 52.2 (47.5-56.2) | 69.2 (64.5-74.0) | 68.4 (64.0-73.0) |
| 2020 | 26.9 (24.6-28.6) | 27.6 (25.1-28.9) | 36.4 (33.8-38.3) | 36.6 (34.2-38.5) | 52.6 (47.7-56.4) | 52.3 (47.6-56.3) | 69.3 (64.5-74.0) | 68.5 (64.1-73.3) |
| 2021 | 26.8 (24.2-28.7) | 27.4 (24.9-28.9) | 36.2 (33.7-38.3) | 36.8 (34.3-38.5) | 52.6 (47.7-56.4) | 52.4 (47.6-56.3) | 69.2 (64.3-74.2) | 68.4 (63.9-73.4) |
| 2022 | 27.1 (24.9-28.7) | 27.3 (25.0-28.8) | 36.2 (33.7-38.2) | 36.5 (34.1-38.5) | 52.5 (47.5-56.3) | 52.4 (47.5-56.3) | 68.8 (64.1-74.0) | 68.0 (63.7-73.2) |
| 2023 | 27.0 (24.6-28.6) | 27.2 (25.0-28.7) | 36.2 (33.7-38.3) | 36.5 (34.1-38.4) | 52.5 (47.4-56.3) | 52.5 (47.5-56.4) | 68.5 (64.0-73.9) | 67.9 (63.7-73.2) |
| 2024 | 27.0 (24.7-28.7) | 27.2 (24.8-28.8) | 36.3 (33.7-38.3) | 36.6 (34.2-38.4) | 52.5 (47.2-56.3) | 52.4 (47.3-56.4) | 68.6 (64.0-74.0) | 68.0 (63.7-73.2) |

Supplementary Table S7: Incidence rate ratio for the sensitivity analysis from 2018-2024 comapring incidence of 18-months duration (original) to 6-months duration (sensitivity)

|  |  |  | Original analysis | | Shorter survival period | |
| --- | --- | --- | --- | --- | --- | --- |
|  |  |  | Males (RR) | Females (RR) | Males (RR) | Females (RR) |
|  |  | 20-29 years | 1.053 (1.038-1.068) | 1.068 (1.055-1.081) | 1.047 (1.033-1.062) | 1.077 (1.065-1.089) |
|  |  | 30-39 years | 1.052 (1.046-1.058) | 1.071 (1.065-1.078) | 1.045 (1.039-1.051) | 1.074 (1.068-1.081) |
|  |  | 40-59 years | 1.029 (1.033-1.061) | 1.061 (1.058-1.063) | 1.036 (1.034-1.038) | 1.058 (1.056-1.061) |
|  |  | 60-79 years | 1.024 (1.022-1.027) | 1.029 (1.026-1.031) | 1.023 (1.021-1.025) | 1.026 (1.023-1.028) |

Supplementary Table S8: Percentage of incident cases only prescribed insulin with a type 2 diabetes code

| sex | age_group | 2017 | 2018 | 2019 | 2020 | 2021 |
| --- | --- | --- | --- | --- | --- | --- |
| Males | 20_29 | 0.92 | 1.32 | 1.38 | 1.02 | 1.09 |
| Males | 30_39 | 0.70 | 0.60 | 0.52 | 1.10 | 0.71 |
| Males | 40_59 | 0.40 | 0.41 | 0.40 | 0.68 | 0.49 |
| Males | 60_79 | 0.50 | 0.39 | 0.39 | 0.53 | 0.56 |
| Females | 20_29 | 0.68 | 0.65 | 0.91 | 0.95 | 0.97 |
| Females | 30_39 | 0.37 | 0.43 | 0.56 | 0.62 | 0.77 |
| Females | 40_59 | 0.35 | 0.31 | 0.30 | 0.41 | 0.32 |
| Females | 60_79 | 0.48 | 0.38 | 0.36 | 0.52 | 0.47 |

Supplementary Table S9: Percentage of people prescribed insulin within six months with a type 2 diabetes code

| sex | age_group | 2017 | 2018 | 2019 | 2020 | 2021 |
| --- | --- | --- | --- | --- | --- | --- |
| Males | 20_29 | 8.26 | 7.93 | 8.76 | 10.71 | 10.14 |
| Males | 30_39 | 4.62 | 4.13 | 4.23 | 6.58 | 6.48 |
| Males | 40_59 | 2.54 | 2.42 | 2.52 | 4.19 | 4.01 |
| Males | 60_79 | 1.83 | 1.59 | 1.58 | 2.55 | 2.71 |
| Females | 20_29 | 7.88 | 7.84 | 7.29 | 8.52 | 8.70 |
| Females | 30_39 | 5.04 | 5.12 | 4.92 | 6.95 | 6.53 |
| Females | 40_59 | 2.11 | 1.89 | 1.87 | 2.92 | 2.85 |
| Females | 60_79 | 1.70 | 1.38 | 1.50 | 2.22 | 2.58 |

Supplementary Table S10: Incidence rate ratios and 95% confidence intervals with original estimates and validated estimates (insulin treated individuals within 6 months excluded) by sex and age from 2018-2024

|  | Original analysis | | Validated type 2 diabetes | |
| --- | --- | --- | --- | --- |
|  | Males (RR) | Females (RR) | Males (RR) | Females (RR) |
| 20-29 years | 1.053 (1.038-1.068) | 1.068 (1.055-1.081) | 1.047 (1.031-1.064) | 1.079 (1.066-1.093) |
| 30-39 years | 1.052 (1.046-1.058) | 1.071 (1.065-1.078) | 1.048 (1.042-1.055) | 1.066 (1.059-1.073) |
| 40-59 years | 1.029 (1.033-1.061) | 1.061 (1.058-1.063) | 1.036 (1.034-1.039) | 1.058 (1.056-1.061) |
| 60-79 years | 1.024 (1.022-1.027) | 1.029 (1.026-1.031) | 1.026 (1.024-1.028) | 1.029 (1.027-1.032) |

Supplementary table S11: cumulative incidence of type 2 diabetes in 2022, 2023 and 2024 combined per 100,000 person years.

The actual value reflects the cumulative incidence observed over the 3-year period, reflecting diagnosis in 2020, 2021 and 2022. The expected, reflects the predicted cumulative incidence based on trends in the preceding years.

|  | | | All ethnicities | White ethnicity | Asian ethnicity | Black ethnicity |
| --- | --- | --- | --- | --- | --- | --- |
| Female | 20-29 years | Expected | 1.06 | 0.84 | 2.12 | 1.73 |
|  |  | Actual | 1.19 | 0.91 | 2.38 | 2.21 |
|  | 30-39years | Expected | 4.2 | 2.9 | 10.1 | 6.8 |
|  |  | Actual | 4.8 | 3.6 | 9.7 | 8.0 |
|  | 40-59 years | Expected | 13.5 | 10.1 | 25.1 | 30.2 |
|  |  | Actual | 14.7 | 11.5 | 27.5 | 27.1 |
|  | 60-79 years | Expected | 18.9 | 16.1 | 37.1 | 42.1 |
|  |  | Actual | 20.3 | 17.6 | 35.6 | 46.5 |
| Male | 20-29 years | Expected | 0.73 | 0.55 | 1.26 | 1.32 |
|  |  | Actual | 0.81 | 0.55 | 1.56 | 1.74 |
|  | 30-39 years | Expected | 4.7 | 2.9 | 12.2 | 7.8 |
|  |  | Actual | 5.2 | 3.4 | 11.6 | 9.4 |
|  | 40-59 years | Expected | 19.4 | 14.4 | 35.3 | 35.0 |
|  |  | Actual | 19.7 | 15.6 | 37.0 | 34.7 |
|  | 60-79 years | Expected | 25.6 | 23.3 | 40.7 | 44.7 |
|  |  | Actual | 28.0 | 25.5 | 41.8 | 34.7 |

Supplementary table S12: BMI within 26 weeks of diagnosis by age and sex with % missing 2011-2024

|  | | Valid BMI with 182 days of diagnosis | | | | BMI for all people Median (IQR) BMI | | | |
| --- | --- | --- | --- | --- | --- | --- | --- | --- | --- |
|  |  | 20-29 yrs | 30-39 yrs | 40-59 yrs | 60-79 yrs | 20-29 years | 30-39 years | 40-59 years | 60-79 years |
| Male | 2011 | 69.6% | 70.5% | 72.2% | 71.5% | 32.9 (27.3-37.6) | 31.0 (26.9-36.2) | 31.1 (27.8-35.3) | 29.8 (27.0-33.0) |
|  | 2012 | 64.6% | 71.3% | 73.0% | 72.7% | 33.5 (28.5-38.3) | 31.0 (27.0-36.4) | 31.1 (27.8-35.4) | 30.0 (27.0-33.2) |
|  | 2013 | 70.1% | 71.1% | 73.5% | 74.3% | 33.2 (28.1-38.5) | 31.2 (27.0-36.4) | 31.2 (27.9-35.5) | 30.0 (27.0-33.4) |
|  | 2014 | 72.6% | 74.0% | 75.9% | 76.1% | 34.7 (28.7-39.8) | 31.1 (27.0-36.8) | 31.3 (27.9-35.6) | 30.0 (27.0-33.6) |
|  | 2015 | 63.7% | 68.3% | 71.4% | 71.1% | 34.0 (29.6-40.7) | 31.3 (27.3-36.6) | 31.3 (27.8-35.8) | 30.1 (27.1-33.7) |
|  | 2016 | 65.5% | 69.7% | 72.3% | 72.1% | 35.2 (29.2-40.2) | 31.6 (27.2-36.8) | 31.4 (27.7-35.8) | 30.2 (27.1-33.8) |
|  | 2017 | 65.0% | 69.5% | 70.5% | 70.1% | 35.7 (29.9-41.7) | 31.7 (27.3-37.2) | 31.5 (27.9-35.9) | 30.3 (27.2-34.0) |
|  | 2018 | 72.9% | 71.7% | 72.0% | 72.0% | 36.4 (31.3-42.0) | 32.0 (27.7-37.6) | 31.6 (27.9-36.1) | 30.4 (27.3-34.1) |
|  | 2019 | 68.8% | 71.6% | 71.7% | 71.5% | 35.6 (31.0-41.1) | 32.3 (27.8-38.3) | 31.4 (27.8-36.0) | 30.4 (27.4-34.1) |
|  | 2020 | 73.5% | 70.5% | 71.7% | 71.6% | 35.5 (29.8-41.7) | 32.3 (28.0-38.3) | 31.5 (27.9-36.0) | 30.4 (27.3-34.2) |
|  | 2021 | 72.0% | 74.2% | 74.7% | 74.5% | 35.8 (29.9-42.8) | 32.7 (28.0-38.6) | 31.5 (27.8-36.0) | 30.5 (27.3-34.3) |
|  | 2022 | 73.1% | 73.3% | 74.1% | 73.3% | 35.5 (30.5-42.1) | 33.1 (28.4-39.2) | 31.9 (28.1-36.6) | 30.8 (27.5-34.8) |
|  | 2023 | 71.6% | 72.3% | 73.0% | 72.4% | 35.5 (30.0-42.1) | 33.4 (28.7-39.4) | 31.8 (28.0-36.5) | 30.9 (27.5-34.9) |
|  | 2024 | 72.0% | 74.5% | 74.7% | 74.3% | 35.2 (29.4-41.9) | 33.3 (28.4-39.5) | 31.7 (28.0-36.4) | 30.8 (27.5-34.8) |
| % change per annum | |  |  |  |  | 0.56% (0.26%-0.86%) | 0.63% (0.55%-0.72%) | 0.17% (0.12%-0.22%) | 0.26% (0.22%-0.3%) |
| Change in kg/m^2^ per yr | |  |  |  |  | 0.20 (0.09-0.30) | 0.20 (0.18-0.23) | 0.05 (0.04-0.07) | 0.08 (0.07-0.09) |
| Female | 2011 | 62.3% | 64.8% | 71.1% | 70.5% | 35.6 (30.0-41.0) | 34.5 (29.0-40.6) | 33.7 (29.1-39.1) | 30.9 (27.1-35.2) |
|  | 2012 | 57.9% | 65.7% | 71.7% | 71.0% | 35.5 (30.0-41.6) | 34.1 (29.1-40.4) | 33.9 (29.2-39.1) | 31.0 (27.2-35.3) |
|  | 2013 | 67.8% | 68.1% | 72.3% | 72.5% | 35.7 (30.3-41.7) | 34.3 (29.0-40.6) | 34.0 (29.3-39.2) | 31.0 (27.3-35.5) |
|  | 2014 | 66.7% | 70.3% | 74.4% | 74.9% | 36.7 (31.0-41.6) | 34.6 (29.1-41.0) | 34.0 (29.5-39.7) | 31.2 (27.4-35.9) |
|  | 2015 | 61.4% | 65.8% | 69.8% | 70.6% | 36.6 (31.6-42.8) | 35.0 (29.7-41.4) | 34.1 (29.5-39.6) | 31.1 (27.3-35.7) |
|  | 2016 | 62.0% | 66.8% | 71.7% | 71.6% | 36.3 (31.2-42.4) | 35.0 (29.9-41.6) | 34.1 (29.5-39.6) | 31.2 (27.4-35.9) |
|  | 2017 | 68.2% | 66.7% | 69.5% | 68.9% | 37.0 (31.1-43.1) | 35.4 (29.9-41.5) | 34.2 (29.7-39.8) | 31.4 (27.5-36.0) |
|  | 2018 | 68.7% | 70.0% | 71.0% | 70.7% | 37.4 (31.8-43.5) | 35.3 (30.1-41.5) | 34.4 (29.8-39.9) | 31.6 (27.7-36.2) |
|  | 2019 | 69.9% | 69.8% | 70.6% | 70.6% | 37.1 (31.2-43.2) | 35.5 (30.1-42.0) | 34.4 (29.7-40.0) | 31.6 (27.7-36.3) |
|  | 2020 | 67.1% | 69.1% | 70.2% | 70.2% | 38.2 (31.9-44.1) | 35.5 (29.9-41.7) | 34.3 (29.7-39.9) | 31.6 (27.5-36.3) |
|  | 2021 | 72.8% | 73.1% | 73.0% | 73.6% | 36.9 (31.1-43.4) | 35.6 (30.1-42.2) | 34.3 (29.7-39.9) | 31.6 (27.6-36.4) |
|  | 2022 | 71.6% | 72.4% | 73.1% | 71.8% | 37.9 (31.7-44.6) | 36.7 (31.2-43.1) | 35.1 (30.2-40.9) | 32.2 (28.0-37.1) |
|  | 2023 | 68.8% | 70.9% | 71.4% | 71.1% | 38.3 (32.3-45.3) | 36.7 (31.1-43.1) | 35.1 (30.2-40.9) | 32.5 (28.2-37.5) |
|  | 2024 | 70.5% | 71.8% | 73.1% | 72.7% | 37.5 (31.2-44.7) | 36.6 (30.6-43.7) | 34.8 (30.0-40.6) | 32.1 (27.9-37.1) |
| % change per annum | |  |  |  |  | 0.52% (0.34%-0.7%) | 0.55% (0.44%-0.66%) | 0.27% (0.19%-0.35%) | 0.35% (0.26%-0.43%) |
| Change in kg/m^2^ per yr | |  |  |  |  | 0.19 (0.13-0.26) | 0.19 (0.15-0.23) | 0.09 (0.06-0.12) | 0.11 (0.08-0.14) |

Supplementary table S13 BMI within 26 weeks of diagnosis by age and sex with imputed missing data, 2011-2024

|  | Year | Median BMI | | | |
| --- | --- | --- | --- | --- | --- |
| Male |  | 20-29 years | 30-39 years | 40-59 years | 60-79 years |
|  | 2011 | 32.8 (27.5- 37.6) | 31.2 (27.2- 36.2) | 31.1 (27.8- 35.4) | 29.7 (26.9- 33) |
|  | 2012 | 34.1 (29.3- 39) | 31.1 (27.3- 36.3) | 31.1 (27.8- 35.4) | 29.9 (27- 33.2) |
|  | 2013 | 33.4 (28.1- 38.3) | 31.3 (27.1- 36.5) | 31.2 (27.8- 35.5) | 30 (27- 33.3) |
|  | 2014 | 34.2 (28.7- 39.5) | 31.2 (27.2- 36.4) | 31.3 (27.9- 35.7) | 30 (27- 33.5) |
|  | 2015 | 35 (30.1- 41.6) | 31.5 (27.5- 36.8) | 31.4 (27.8- 35.9) | 30 (26.9- 33.6) |
|  | 2016 | 34.8 (29.2- 39.8) | 31.8 (27.4- 36.9) | 31.4 (27.8- 35.8) | 30.1 (27- 33.8) |
|  | 2017 | 35.5 (29.9- 41.2) | 31.9 (27.6- 37.3) | 31.5 (27.9- 35.9) | 30.1 (27.1- 33.9) |
|  | 2018 | 36 (30.8- 41.8) | 32.1 (28- 37.6) | 31.6 (28- 36.2) | 30.4 (27.2- 34.1) |
|  | 2019 | 35.4 (30.7- 40.3) | 32.4 (28- 38.2) | 31.5 (27.9- 36.1) | 30.3 (27.2- 34) |
|  | 2020 | 35.6 (30.3- 41.7) | 32.5 (28.3- 38.3) | 31.6 (28- 36.1) | 30.3 (27.2- 34.1) |
|  | 2021 | 35.4 (29.9- 42.1) | 32.9 (28.2- 38.7) | 31.6 (27.9- 36.1) | 30.4 (27.1- 34.2) |
|  | 2022 | 35.6 (30.5- 42.2) | 33.4 (28.7- 39.2) | 32 (28.1- 36.7) | 30.8 (27.4- 34.8) |
|  | 2023 | 35.6 (30.1- 42.2) | 33.5 (28.9- 39.3) | 31.8 (28- 36.6) | 30.8 (27.4- 34.9) |
|  | 2024 | 35 (29.8- 41.3) | 33.5 (28.6- 39.5) | 31.8 (28- 36.5) | 30.7 (27.4- 34.7) |
| Change per annum (%) | | 0.49 (0.24-0.74) | 0.64 (0.56—0.73) | 0.19 (0.14-0.23) | 0.25(0.20-0.31) |
| Change per year (kg/m^2^) | | 0.17 (0.08-0.26) | 0.21(0.18-0.24) | 0.06 (0.04-0.07) | 0.08 (0.06-0.09) |
|  | 2011 | 35.8 (30.8- 42) | 34.6 (29.1- 40.7) | 33.7 (29.2- 39) | 31 (27.3- 35.2) |
|  | 2012 | 36 (30.7- 41.8) | 34.4 (29.4- 40.5) | 33.8 (29.2- 39) | 31 (27.3- 35.2) |
|  | 2013 | 35.6 (30.5- 41) | 34.5 (29.5- 40.6) | 33.9 (29.3- 39.1) | 31 (27.4- 35.4) |
|  | 2014 | 37 (31- 41.7) | 34.6 (29.3- 40.8) | 34 (29.4- 39.5) | 31.1 (27.5- 35.7) |
|  | 2015 | 37 (32.2- 43.9) | 35 (30- 41.1) | 34 (29.5- 39.5) | 31.1 (27.3- 35.6) |
|  | 2016 | 36.9 (31.2- 43.1) | 35 (30- 41.6) | 34.2 (29.6- 39.7) | 31.3 (27.6- 35.9) |
|  | 2017 | 37.2 (31.6- 43) | 35.4 (30- 41.4) | 34.2 (29.7- 39.7) | 31.4 (27.6- 36) |
|  | 2018 | 37.5 (32.3- 43.6) | 35.3 (30.2- 41.4) | 34.3 (29.7- 39.8) | 31.6 (27.8- 36.2) |
|  | 2019 | 37.5 (31.7- 43.3) | 35.7 (30.3- 42.1) | 34.4 (29.7- 39.9) | 31.6 (27.8- 36.3) |
|  | 2020 | 38.4 (32.2- 44.1) | 35.6 (30.3- 41.9) | 34.2 (29.7- 39.9) | 31.6 (27.7- 36.3) |
|  | 2021 | 36.7 (31- 43) | 35.9 (30.4- 42.3) | 34.3 (29.7- 39.8) | 31.6 (27.7- 36.4) |
|  | 2022 | 37.7 (31.8- 44.5) | 36.7 (31.2- 43) | 35 (30.1- 40.8) | 32.2 (28.1- 37.1) |
|  | 2023 | 38.8 (32.7- 45.7) | 37 (31.4- 43.2) | 35.1 (30.2- 40.8) | 32.5 (28.3- 37.4) |
|  | 2024 | 37.4 (31.2- 44.6) | 36.8 (30.9- 43.8) | 34.7 (30- 40.5) | 32.1 (28- 37) |
| Change per annum (%) | | 0.47 (0.24-0.69) | 0.56 (0.46-0.67) | 0.26 (0.19-0.34) | 0.34 (0.25-0.42) |
| Change per year (kg/m^2^) | | 0.17(0.09-0.26) | 0.20 (0.16-0.24) | 0.09 (0.06-0.12) | 0.11 (0.08-0.13) |

Supplementary table S14: BMI within 26 weeks of diagnosis by age and sex for people of White ethnicity, 2011-2024

|  | Year | Median BMI (inter-quartile range) | | | |
| --- | --- | --- | --- | --- | --- |
|  |  | 20-29 years | 30-39 years | 40-59 years | 60-79 years |
|  | 2011 | 35.5 (30.5- 40.3) | 33.9 (29.9- 38.9) | 32.1 (28.7- 36.3) | 30 (27- 33.3) |
|  | 2012 | 36.6 (32- 40.4) | 34 (30- 39.1) | 32.1 (28.7- 36.4) | 30 (27- 33.5) |
|  | 2013 | 35.5 (30.8- 40.3) | 34.2 (30- 39.8) | 32.2 (28.8- 36.6) | 30.1 (27.1- 33.7) |
|  | 2014 | 36.1 (31.1- 41) | 34 (29.6- 39.5) | 32.4 (29- 36.8) | 30.2 (27.2- 33.9) |
|  | 2015 | 38.1 (33.2- 44) | 34.7 (30- 39.7) | 32.5 (29- 37) | 30.2 (27.3- 34) |
|  | 2016 | 36.7 (31.4- 42.1) | 34.7 (30.1- 40) | 32.6 (29- 37) | 30.4 (27.4- 34.2) |
| Male | 2017 | 38.4 (33.5- 44.2) | 35.3 (30.6- 40.5) | 32.9 (29.2- 37.3) | 30.6 (27.4- 34.3) |
|  | 2018 | 38.3 (33.3- 43.7) | 35.4 (30.8- 40.7) | 33.1 (29.3- 37.6) | 30.7 (27.6- 34.5) |
|  | 2019 | 37.3 (33.1- 42.7) | 35.7 (30.9- 41.4) | 32.9 (29.1- 37.6) | 30.7 (27.6- 34.5) |
|  | 2020 | 37.6 (32.1- 43.7) | 35.6 (31.1- 41.4) | 33 (29.3- 37.6) | 30.7 (27.6- 34.5) |
|  | 2021 | 38.3 (32.5- 45) | 36 (31.1- 42) | 33 (29.3- 37.7) | 30.8 (27.7- 34.7) |
|  | 2022 | 37.5 (32.3- 44.1) | 36.3 (31.5- 42.2) | 33.5 (29.7- 38.4) | 31.2 (27.9- 35.3) |
|  | 2023 | 38.5 (33.1- 45) | 36.6 (31.7- 42) | 33.4 (29.5- 38.3) | 31.4 (28- 35.4) |
|  | 2024 | 38.3 (32.5- 44.6) | 37.1 (32.1- 43) | 33.5 (29.6- 38.3) | 31.2 (28- 35.3) |
|  |  |  |  |  |  |
|  | 2011 | 37.7 (32.4- 44) | 37.3 (32.3- 43.6) | 35 (30.4- 40.5) | 31.1 (27.6- 35.5) |
|  | 2012 | 37.9 (32.7- 43.3) | 37.4 (32.1- 43.3) | 35 (30.6- 40.5) | 31.2 (27.6- 35.7) |
|  | 2013 | 37.8 (32.2- 43) | 38 (32.5- 43.7) | 35.2 (30.5- 40.6) | 31.4 (27.7- 35.8) |
|  | 2014 | 38.2 (33.2- 43) | 37.9 (32.6- 43.9) | 35.5 (31- 41) | 31.6 (27.9- 36) |
|  | 2015 | 39.6 (34- 46.8) | 38.4 (33.2- 44.5) | 35.4 (30.9- 41.2) | 31.4 (27.6- 36) |
|  | 2016 | 39.3 (33.7- 46.3) | 38.4 (32.7- 44.3) | 35.7 (30.9- 41) | 31.7 (27.9- 36.3) |
| Fenale | 2017 | 38.8 (33.6- 44.8) | 38.7 (32.9- 44.8) | 35.8 (31.1- 41.3) | 31.9 (28- 36.5) |
|  | 2018 | 39.2 (34.3- 45.5) | 38.3 (33.3- 44.7) | 36 (31.2- 41.6) | 32.2 (28.2- 36.7) |
|  | 2019 | 39 (33.7- 45.2) | 39.3 (33.9- 44.9) | 36.1 (31.3- 41.7) | 32.2 (28.2- 36.9) |
|  | 2020 | 40.1 (35.4- 45.8) | 39.2 (34- 45.3) | 35.9 (31.2- 41.7) | 32.1 (28.2- 36.8) |
|  | 2021 | 38.6 (33.2- 45.4) | 39.5 (34- 45.5) | 36.1 (31.4- 41.6) | 32.1 (28.1- 37) |
|  | 2022 | 40 (34.5- 46.4) | 40 (34.4- 46.2) | 36.8 (31.9- 42.5) | 32.9 (28.7- 37.9) |
|  | 2023 | 41.5 (35.7- 48.1) | 40 (34.8- 46) | 37 (32.1- 42.9) | 33.2 (29- 38.2) |
|  | 2024 | 40.7 (34.7- 47.2) | 40.6 (34.8- 46.7) | 36.7 (31.8- 42.6) | 32.9 (28.7- 37.8) |

Supplementary table S15: BMI within 26 weeks of diagnosis by age and sex for people of Asian ethnicity, 2011-2024

|  | Year | Median BMI (inter-quartile range) | | | |
| --- | --- | --- | --- | --- | --- |
|  |  | 20-29 years | 30-39 years | 40-59 years | 60-79 years |
|  | 2011 | 29 (25.2- 33.5) | 28.4 (25.3- 32.3) | 28 (25.2- 31) | 27 (24.7- 29.8) |
|  | 2012 | 30 (26- 33.7) | 28.4 (25.4- 31.8) | 27.9 (25.3- 31.1) | 27.1 (24.7- 30) |
|  | 2013 | 29 (26.2- 33.4) | 28.4 (25.4- 32.3) | 28 (25.3- 31) | 26.9 (24.5- 29.8) |
|  | 2014 | 30.1 (26.3- 36.5) | 28.7 (25.7- 32.6) | 28 (25.5- 31.2) | 27.3 (25- 30) |
|  | 2015 | 31.1 (27.2- 35) | 28.9 (25.8- 32.9) | 28.1 (25.4- 31.3) | 27.3 (24.8- 30.1) |
|  | 2016 | 31 (27.6- 35.8) | 29.4 (25.8- 33.3) | 28 (25.5- 31.1) | 27.3 (24.7- 30.3) |
| Male | 2017 | 30.9 (26.9- 35.2) | 29 (25.9- 33) | 28.2 (25.7- 31.4) | 27.4 (25- 30.3) |
|  | 2018 | 32.8 (29- 37.2) | 29.2 (26- 33.3) | 28.3 (25.6- 31.6) | 27.4 (24.9- 30.4) |
|  | 2019 | 31.9 (28.1- 35.8) | 29.3 (26.2- 33.5) | 28.4 (25.7- 31.7) | 27.7 (25.1- 30.5) |
|  | 2020 | 32.3 (28.1- 36.9) | 29.6 (26.2- 33.3) | 28.4 (25.8- 31.8) | 27.5 (25.1- 30.5) |
|  | 2021 | 31.5 (27.5- 36.1) | 29.7 (26.2- 34) | 28.3 (25.6- 31.9) | 27.5 (25- 30.3) |
|  | 2022 | 32.2 (27.5- 39) | 30 (26.5- 34) | 28.7 (25.9- 32.1) | 27.5 (25.1- 30.5) |
|  | 2023 | 32.5 (28.4- 36.5) | 29.9 (26.6- 33.9) | 28.4 (25.6- 31.8) | 27.3 (24.9- 30.4) |
|  | 2024 | 32.3 (27.7- 36.9) | 29.8 (26.5- 34.4) | 28.5 (25.8- 32) | 27.2 (24.9- 30.3) |
|  |  |  |  |  |  |
|  | 2011 | 32 (27.3- 37) | 30.2 (27- 34.8) | 30.1 (27- 34.1) | 28.9 (26- 32.5) |
|  | 2012 | 31 (27.7- 35.6) | 30.7 (26.9- 35) | 29.9 (26.9- 33.8) | 28.9 (25.6- 32.5) |
|  | 2013 | 32 (27.6- 36.4) | 30.6 (27- 35.5) | 30.1 (27.1- 34) | 29 (26.1- 32.5) |
|  | 2014 | 30.5 (26.6- 36.3) | 31.3 (27.3- 35.2) | 30.1 (27- 34.3) | 29 (26- 32.7) |
|  | 2015 | 33.3 (28.2- 37.7) | 31.2 (27.5- 35.7) | 30.5 (27.3- 34.5) | 29 (26- 32.5) |
|  | 2016 | 32.5 (28.3- 36.3) | 31.6 (27.8- 35.7) | 30.7 (27.3- 34.6) | 28.9 (25.9- 32.9) |
| Fenale | 2017 | 32.3 (28- 37.6) | 31.1 (27.4- 36) | 30.7 (27.3- 34.6) | 29.1 (26- 32.9) |
|  | 2018 | 34.3 (29.4- 38.9) | 31.2 (27.7- 35.9) | 30.5 (27.3- 34.6) | 29.4 (26- 33.2) |
|  | 2019 | 33.5 (28- 38.9) | 31.9 (28- 36.2) | 30.7 (27.3- 34.9) | 29 (26.1- 32.7) |
|  | 2020 | 33.4 (29- 38.4) | 31.5 (27.6- 35.9) | 30.9 (27.6- 34.9) | 29 (25.9- 32.5) |
|  | 2021 | 34.3 (28.7- 39.1) | 31.6 (27.7- 36.3) | 30.8 (27.4- 34.9) | 29 (26- 32.8) |
|  | 2022 | 33.4 (29.4- 39.8) | 32.2 (28.1- 36.7) | 31.1 (27.8- 35.3) | 29.2 (26.1- 33) |
|  | 2023 | 33.8 (29- 38.6) | 32 (28.2- 36.9) | 30.9 (27.5- 35.3) | 28.9 (25.8- 32.5) |
|  | 2024 | 32.8 (28.4- 38.1) | 31.9 (27.9- 36.6) | 30.8 (27.5- 35.1) | 28.9 (25.7- 32.5) |

Supplementary table S16: BMI within 26 weeks of diagnosis by age and sex for people of Black ethnicity, 2011-2024

|  | Year | Median BMI (inter-quartile range) | | | |
| --- | --- | --- | --- | --- | --- |
|  |  | 20-29 years | 30-39 years | 40-59 years | 60-79 years |
|  | 2011 | 31.1 (25.2- 36.2) | 29 (26- 33.3) | 29.4 (26.4- 33) | 28.2 (25.5- 31) |
|  | 2012 | 32.1 (29.2- 35.2) | 29.8 (26- 33.8) | 29.6 (26.6- 33) | 28.5 (26- 31.6) |
|  | 2013 | 32 (28- 37.6) | 29.7 (26.1- 34) | 29.7 (26.8- 33.1) | 28.2 (25.6- 31.7) |
|  | 2014 | 34.3 (29- 40) | 30 (26.8- 33.5) | 29.8 (26.6- 33.2) | 29 (26.2- 32) |
|  | 2015 | 32.9 (29.7- 40.3) | 30.3 (26.5- 35.5) | 30 (27- 33.5) | 28.9 (26.1- 32.3) |
|  | 2016 | 33.2 (27.7- 39.7) | 30.9 (27.4- 35.8) | 30 (26.8- 33.6) | 29 (26.1- 31.9) |
| Male | 2017 | 31.3 (27.8- 38.3) | 29.6 (26.5- 34.5) | 29.9 (26.9- 33.4) | 28.5 (25.9- 32) |
|  | 2018 | 32.9 (27- 36.8) | 30.6 (26.9- 35.4) | 30 (27- 33.8) | 28.7 (26.1- 32) |
|  | 2019 | 34.3 (29.3- 39.3) | 30.5 (26.7- 34.7) | 30 (27- 33.7) | 28.7 (26.2- 32) |
|  | 2020 | 33.7 (27.5- 40.1) | 31.2 (27- 36.2) | 30 (27.1- 33.9) | 28.9 (26.1- 31.9) |
|  | 2021 | 34.4 (29.3- 37.3) | 31 (26.8- 36.3) | 29.9 (26.8- 33.7) | 28.8 (26.1- 32) |
|  | 2022 | 34.3 (30.5- 39.6) | 30.8 (26.8- 36.6) | 30.1 (27- 34) | 29.4 (26.5- 32.5) |
|  | 2023 | 34.5 (29.3- 38.6) | 31.4 (27.6- 36.3) | 30.3 (27- 34.1) | 29.2 (26.4- 32.8) |
|  | 2024 | 33 (29.5- 36.5) | 31 (27.2- 36.4) | 30.1 (26.9- 34) | 29.2 (26.5- 32.6) |
|  |  |  |  |  |  |
|  | 2011 | 33.5 (30- 39.1) | 32.1 (28.4- 38) | 32.7 (28.8- 37.3) | 30.8 (27.4- 35) |
|  | 2012 | 34.6 (30.7- 41.5) | 33 (29- 37.3) | 32.6 (28.4- 37) | 30.6 (27.2- 34.1) |
|  | 2013 | 35.3 (30.2- 39.4) | 33.4 (29- 37.3) | 33.1 (29.4- 38) | 30.8 (27.3- 34.7) |
|  | 2014 | 38 (30.1- 41.8) | 32.6 (28.5- 37.5) | 33 (29- 37.4) | 31 (27.4- 35.1) |
|  | 2015 | 36.9 (31.5- 44.7) | 35.1 (30.1- 39.8) | 33.4 (29- 38.1) | 30.8 (27.5- 35.1) |
|  | 2016 | 32.5 (28- 40.3) | 34.9 (28.8- 39.8) | 33.4 (29.5- 38.3) | 31.2 (27.8- 35.7) |
| Fenale | 2017 | 37.4 (29.8- 44.2) | 34.6 (30.6- 40.2) | 33.6 (29.5- 38.4) | 31.6 (27.9- 35.9) |
|  | 2018 | 37.1 (31.5- 43.2) | 34.4 (29.1- 38.9) | 33.5 (29.5- 38.1) | 31.2 (27.9- 35.3) |
|  | 2019 | 36.4 (31.3- 41.8) | 33.3 (29.3- 38.8) | 33.5 (29.6- 38) | 31.6 (28.3- 35.7) |
|  | 2020 | 36.3 (31.1- 44) | 34.5 (29.6- 40.7) | 33.2 (29.7- 38.1) | 31.5 (27.8- 35.7) |
|  | 2021 | 34.8 (29.4- 41.2) | 34.8 (30.5- 40.9) | 33.5 (29.5- 38.3) | 31.4 (27.9- 36) |
|  | 2022 | 35.8 (30.9- 44.2) | 35.3 (30- 40.1) | 33.8 (29.8- 38.9) | 32.1 (28.4- 36.8) |
|  | 2023 | 38.1 (32.7- 44.1) | 34.7 (29.9- 40.5) | 33.8 (29.9- 38.8) | 32.5 (28.4- 37) |
|  | 2024 | 36.7 (31.3- 41.2) | 35 (30.5- 40.8) | 33.7 (29.7- 38.5) | 32 (28.2- 36.5) |
